# Regulation of diabetic wound healing by the gut–liver axis via immunometabolic reprogramming of macrophages: from systemic pre-programming to targeted therapy

**DOI:** 10.3389/fimmu.2026.1991888

**Published:** 2026-10-30

**Authors:** Shengyi Zhao, Enyu Yu, Fenglin Wu, Yang Zhang, Xue Zhao, Xueping Wu

**Affiliations:** 1 School of Clinical Medicine, Shanghai University of Medicine & Health Sciences, Shanghai, China; 2 Laboratory of Tumor Molecular Imaging, Pudong Gongli Hospital, Shanghai University of Medicine & Health Sciences, Shanghai, China

**Keywords:** diabetic wounds, exosomes, gut microbiota, gut–liver axis, immunometabolic reprogramming, M1-like/M2-like macrophages, macrophage polarization, systemic pre-programming

## Abstract

Diabetic wounds, particularly diabetic foot ulcers, remain a major clinical challenge due to their recalcitrance to healing and limited response to conventional therapies. Successful wound healing critically depends on timely macrophage phenotypic switching from a pro-inflammatory M1-like to a reparative M2-like state; however, the diabetic milieu—characterized by hyperglycemia and advanced glycation end products (AGEs)—impedes this transition, establishing macrophage dysfunction as a hallmark of chronic non-healing wounds. We propose a conceptual framework in which gut–liver axis dysfunction in diabetes does not merely act through direct delivery of metabolites to the wound bed, but may contribute to a state of systemic immunometabolic dysregulation that “pre-programs” bone-marrow-derived monocytes toward a pro-inflammatory M1-like bias before they reach the wound site. We discuss three potential components of this pre-programming: (i) the cellular targets—hematopoietic stem and progenitor cells and circulating monocyte precursors; (ii) the candidate signaling mediators—gut-derived lipopolysaccharide (LPS), inflammatory cytokines, and exosomes acting via the toll-like receptor 4 (TLR4)/NF-κB pathway; and (iii) the proposed molecular imprint—persistent modifications of histone marks such as H3K4me3 and H3K27ac at promoters of pro-inflammatory genes, accompanied by a metabolic shift toward aerobic glycolysis. Importantly, direct evidence that gut–liver-derived exosomes epigenetically remodel hematopoietic progenitors in diabetic wound healing is currently lacking, and this remains a hypothesis generated by integrating separate lines of evidence. Reduced gut-derived short-chain fatty acids (SCFAs) and disrupted hepatic bile acid metabolism may further exacerbate immune dysregulation within the wound microenvironment. On this basis, targeting the gut–liver axis to restore macrophage homeostasis has emerged as a promising therapeutic strategy. We summarize current evidence on probiotics, fecal microbiota transplantation, bile acid modulation, exosome-based delivery, and metabolic interventions, and discuss how these approaches may restore gut–liver axis homeostasis and reprogram macrophage phenotypes to promote wound healing. We clearly indicate the evidence level of each proposed intervention—human clinical trial, animal model, *in vitro*, or not yet tested. Looking ahead, the integration of multi-omics technologies with smart nanodelivery systems and personalized medicine strategies is expected to accelerate the translation of this regulatory network from basic research to clinical application, providing new conceptual and therapeutic avenues for the management of diabetic wounds.

## 1 Introduction

Chronic non-healing wounds caused by Diabetes Mellitus (DM), particularly diabetic foot ulcers, are among the leading causes of disability, mortality, and increased healthcare burden in affected patients. Their underlying pathophysiology is highly complex, and conventional treatments are often of limited efficacy because they fail to reverse the profound systemic derangements that drive the disease. In contrast to physiological wound healing, diabetic wounds remain trapped in a state of excessive and prolonged inflammation and fail to progress efficiently to the proliferative and tissue-remodeling phases. Beyond local neurovascular pathology, systemic immunometabolic dysregulation is increasingly recognized as a key driving force underlying these impaired healing responses (1–3).

In this process, macrophages act as key regulators of wound repair, and their functional state directly influences both the resolution of inflammation and the regeneration of tissue. Under diabetic pathological conditions, the transition of macrophages from a pro-inflammatory (M1-like) to a reparative (M2-like) phenotype is impaired, causing them to remain predominantly in an M1-like state, which exacerbates local inflammation and hinders the wound healing process (4). It should be noted that the M1-like/M2-like nomenclature is a simplification; macrophages exist along a continuous activation spectrum, and diabetic wounds likely contain mixed and plastic phenotypes. In this Review, we use “M1-like” and “M2-like” to denote the extreme ends of this spectrum. Notably, macrophage dysfunction does not arise solely from local hyperglycemia or the action of advanced glycation end products (AGEs) within the wound; rather, even before they infiltrate the wound, macrophages are already subjected to systemic regulation by the whole-body metabolic and immune milieu (3, 5).

In recent years, the concept of the “gut–liver axis” has provided important clues for understanding this systemic preprogramming. The gut–liver axis refers to a bidirectional regulatory network linking the gut microbiota, intestinal barrier, and liver via the portal circulation, bile acid recycling, and diverse metabolic and inflammatory signals, and it serves as a critical bridge between nutrient metabolism and systemic immune responses (6). In metabolic diseases such as type 2 diabetes and obesity, gut microbiota dysbiosis and impaired barrier function can promote the translocation of bacterial components, such as lipopolysaccharide (LPS), and microbial metabolites into the portal circulation, thereby triggering metabolic endotoxemia. This process activates hepatic Kupffer cells and hepatocytes, promoting the development of metabolic dysfunction-associated steatotic liver disease (MASLD) and systemic low-grade chronic inflammation (7, 8).

It is important to distinguish established findings from the hypothesis proposed in this Review. Bone marrow priming in diabetes has been demonstrated by the Gallagher laboratory and by studies on trained immunity, which show that diabetic conditions can alter the epigenetic and functional state of bone marrow progenitors and circulating monocytes (3, 9). In contrast, the possibility that gut–liver axis-derived signals—particularly exosomes—contribute to this priming in the context of diabetic wound healing has not yet been directly demonstrated. The present Review therefore integrates separate lines of evidence to propose a conceptual framework rather than to assert a proven mechanism. Where direct evidence is lacking, we explicitly state that the mechanism remains hypothetical and identify experimental approaches that could test it.

This persistent metabolic inflammation not only alters hepatic glucose–lipid metabolism and bile acid synthesis, but also remodels bone marrow hematopoiesis and the epigenetic landscape and polarization bias of peripheral monocytes through cytokines, lipid mediators, and exosomes. Consequently, we propose that the gut–liver axis may contribute to a systemic pro-inflammatory “set point” that potentially primes circulating monocytes, which then, upon recruitment to the wound and encounter with local hyperglycemic and hypoxic stimuli, are predisposed to lock into the M1-like phenotype. Meanwhile, diabetes-associated dysregulation of hepatic lipid metabolism is often accompanied by impaired synthesis of specialized pro-resolving lipid mediators, such as resolvins and protectins (10), together with reduced levels of gut-derived immunoregulatory metabolites, including short-chain fatty acids (SCFAs) (11) deficits further compromise the ability to restore immune homeostasis and promote resolution of inflammation within the wound microenvironment.

Therefore, elucidating in depth the molecular mechanisms by which the gut–liver axis may contribute to regulating macrophage function through metabolic–immune networks and thereby influence diabetic wound repair will not only advance our understanding of the systemic causes underlying impaired healing, but also provide a theoretical basis for the development of novel targeted therapeutic strategies. This review aims to systematically summarize the roles and mechanisms of the gut–liver axis–macrophage regulatory network in diabetic wound repair and, based on current evidence, to explore potential therapeutic strategies that restore macrophage homeostasis and promote wound healing through multiple approaches, including probiotics, bile acid modulators, exosome-based therapies, and metabolic interventions, with the ultimate goal of offering new perspectives and avenues for clinical translation in the management of diabetic wounds.

## 2 Current advances in research

### 2.1 Roles and regulatory mechanisms of macrophages in diabetic wound healing

Macrophages play a pivotal role in diabetic wound repair, and their dysfunction is one of the major contributors to delayed and chronic wound healing in diabetes. Recent advances have largely focused on macrophage polarization states, the underlying regulatory mechanisms, and therapeutic strategies that target macrophages to enhance wound healing in diabetes.

In physiological wound healing, macrophages exhibit marked plasticity, and their temporal transition from a pro-inflammatory (M1-like) to a reparative (M2-like) phenotype is central to orchestrating inflammation resolution and tissue regeneration. Early in this process, M1-like macrophages eliminate pathogens and initiate immune responses by secreting factors such as tumor necrosis factor-alpha (TNF-α) and interleukin-1 beta (IL-1β), whereas at later stages, M2-like macrophages promote angiogenesis and extracellular matrix remodeling via interleukin-10 (IL-10), transforming growth factor-beta (TGF-β), and related mediators (12, 13). However, the diabetic pathological milieu (including hyperglycemia, oxidative stress, and AGEs) disrupts this balance, leading to pathological persistence of the M1-like phenotype and impaired polarization toward M2-like (14). This defect in phenotypic switching traps the wound in a state of chronic low-grade inflammation, severely impairing angiogenesis and extracellular matrix deposition and ultimately resulting in stalled healing. Consequently, restoring the dynamic balance between M1-like and M2-like functional states has emerged as a key therapeutic strategy for improving outcomes in diabetic wound healing.

The failure of macrophages to transition from a pro-inflammatory to a pro-repair phenotype is a key pathological hallmark of non-healing diabetic wounds and is underpinned by profound cellular and molecular abnormalities. Hyperglycemia and metabolic dysregulation are central drivers of this imbalance: chronic hyperglycemia not only directly impairs macrophage chemotaxis and phagocytic capacity, but also fundamentally suppresses their normal transition from the M1-like to the M2-like phenotype, resulting in prolonged retention of M1-like macrophages within the wound and perpetuation of a deleterious cycle of chronic low-grade inflammation.

At the level of signaling pathways, classical inflammatory cascades such as NF-κB play pivotal roles. Persistent inflammatory stimuli in the diabetic microenvironment lead to excessive activation of the NF-κB pathway, thereby consolidating the pro-inflammatory M1-like phenotype at the transcriptional level and antagonizing the reparative gene programs required for M2-like polarization.

Epigenetic regulation adds an additional layer of fine-tuned control: mechanisms such as histone modifications dynamically alter chromatin accessibility, profoundly influencing the expression of genes involved in macrophage polarization and thereby modulating their responsiveness to microenvironmental cues (15). In addition, defective efferocytosis represents a critical link between early inflammation and subsequent failure of tissue repair. Efferocytosis—the macrophage-mediated clearance of apoptotic cells, which in the wound includes apoptotic neutrophils as well as apoptotic cells of other lineages, such as fibroblasts, keratinocytes, and lymphocytes—is not merely a disposal mechanism but also a potent physiological cue for the transition from the M1-like to the M2-like phenotype (16, 17). However, in diabetic wounds, excessive formation of neutrophil extracellular traps and impairment of downstream signaling pathways, such as phosphoinositide 3-kinase (PI3K)/Rac1, markedly compromise macrophage efferocytic capacity (18), thereby interrupting the natural course of phenotypic switching and resolution of inflammation.

Given the central role of imbalanced macrophage polarization in non-healing diabetic wounds, targeted modulation of macrophage phenotype and function has emerged as a highly promising therapeutic approach. Current strategies are evolving from conventional cell-based therapies toward precise interventions that exploit cell-derived products. In terms of cell therapy, mesenchymal stem cells (MSCs) have been widely used owing to their potent paracrine and immunomodulatory capacities; in a preclinical study, human umbilical cord MSCs engineered to overexpress IL-4, IL-10 and IL-13 promoted M2-like polarization of murine macrophages *in vitro* and improved healing in a diabetic wound model (19). The research frontier has further focused on bioactive carriers secreted by cells. For example, macrophage-derived extracellular vesicles (EVs) are naturally enriched with diverse bioactive molecules capable of orchestrating immune responses, promoting angiogenesis and cellular proliferation, thereby offering a novel nanoscale therapeutic option (20) (characterized *in vitro* as large extracellular vesicles from human regulatory macrophages, without *in vivo* testing). Similarly, exosomes derived from platelet-rich plasma have also been shown to effectively drive macrophage polarization toward a reparative M2-like phenotype, thereby accelerating the healing of diabetic foot ulcers (21) (demonstrated in animal models of diabetic wounds). For example, apoptotic bodies derived from adipose-derived stem cells exhibit marked immunomodulatory and tissue-regenerative potential (22) (demonstrated in a diabetic rat wound model, in which the effect was mediated by miR-20a-5p/JAK-STAT signaling), whereas their exosomes can deliver molecules such as miR-146a-5p to downstream targets including JAZF1, thereby enhancing angiogenic activity *in vitro* (23) (no *in vivo* wound model). Together, these strategies constitute a multi-tiered, stepwise therapeutic framework, indicating that the treatment of diabetic wounds is evolving toward increasingly refined and targeted interventions.

In summary, dysregulated macrophage polarization represents a central pathological node in non-healing diabetic wounds. The diabetic microenvironment, through sustained activation of NF-κB and related signaling pathways, drives aberrant activation of M1-like macrophages while concurrently suppressing M2-like polarization and function, thereby locking the wound into a vicious cycle of chronic inflammation and stalled repair. Current therapeutic strategies focus on the precise reprogramming of macrophage trajectories, particularly through the use of mesenchymal stem cells and their exosomes, as well as other bioactive carriers, with the aim of reconstructing the healing microenvironment in a spatiotemporally controlled manner. This shift signifies a profound transition in therapeutic concepts from local wound care to systemic regulation of immune homeostasis, and it offers new perspectives for understanding the links between diabetic wounds and global metabolic dysregulation (**Figure 1**).

**Figure 1:**
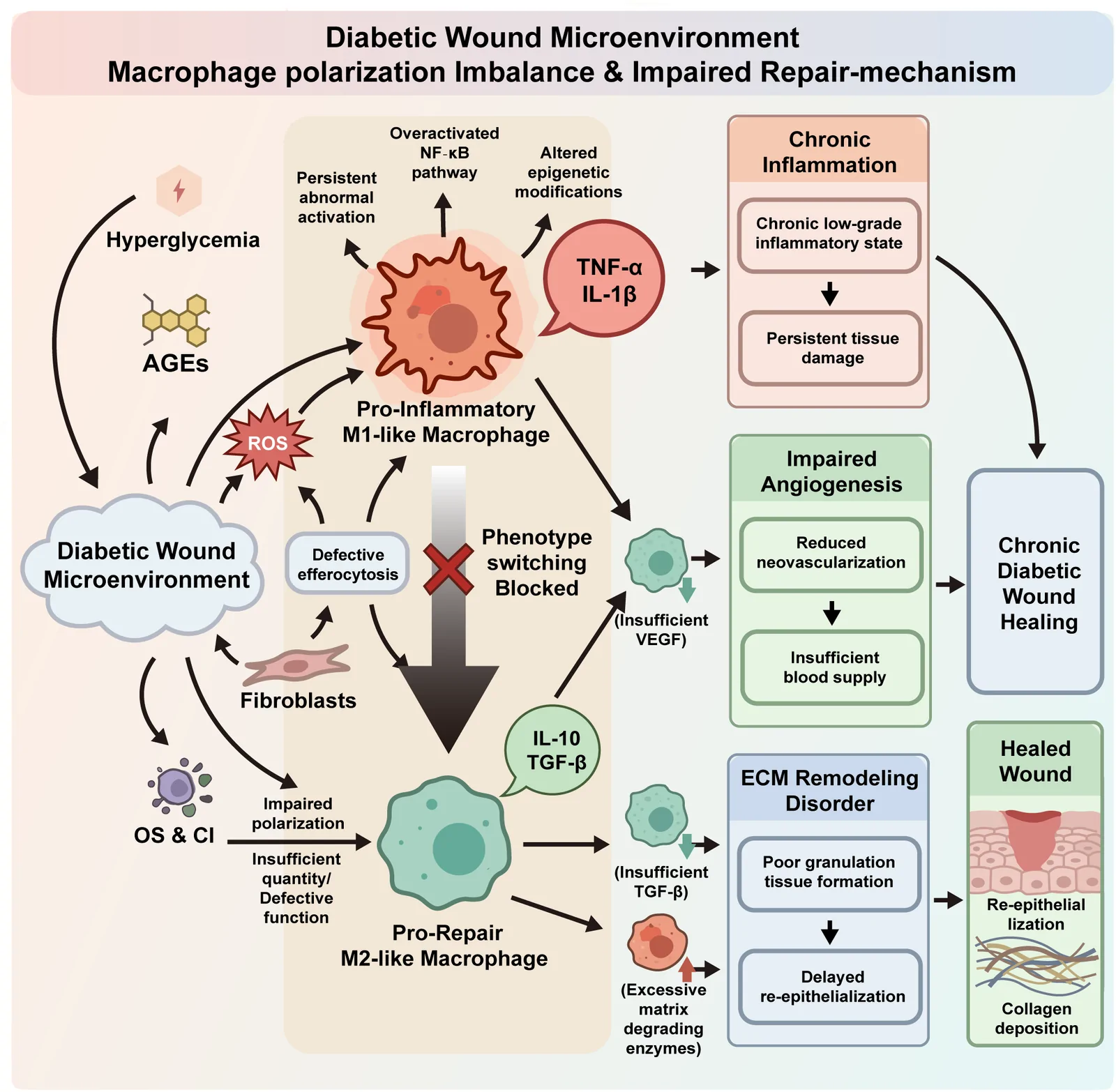
Schematic illustration of imbalanced macrophage polarization and impaired repair mechanisms in the diabetic wound microenvironment. Within the diabetic wound microenvironment, shaped by hyperglycemia, AGEs, and ROS, macrophages are persistently driven toward a pro-inflammatory M1-like phenotype, primarily through excessive activation of the NF-κB signaling pathway, resulting in abundant release of pro-inflammatory mediators such as TNF-α and IL-1β. At the same time, the temporal transition from M1-like to the reparative M2-like phenotype is markedly impaired, as evidenced by a reduction in M2-like macrophage numbers and a deficiency of reparative factors, including IL-10 and TGF-β. This imbalance in immune polarization ultimately leads to delayed and chronic wound healing through three major mechanisms (1): sustaining and amplifying local chronic inflammation (2); suppressing angiogenesis and re-epithelialization; and (3) disrupting normal deposition and remodeling of the extracellular matrix (ECM). AGEs, advanced glycation end products; ROS, reactive oxygen species; ECM, extracellular matrix; NF-κB, Nuclear Factor kappa-B; TNF-α, Tumor Necrosis Factor-alpha; IL-1β, Interleukin-1 beta; OS, Oxidative Stress; CI, Chronic Inflammation; VEGF, Vascular Endothelial Growth Factor; IL-10, Interleukin-10; TGF-β, Transforming Growth Factor-beta.

### 2.2 Potential links between the gut–liver axis and diabetic wound repair

To date, no study has simultaneously validated the interrelationships among gut microbiota, liver inflammation, and wound macrophage phenotypes within a single experimental system; therefore, the framework described in this section is a speculative one based on existing indirect evidence. We hypothesize that, in the context of diabetes, the gut-liver axis may participate in the regulation of wound healing through the three interrelated mechanisms shown in **Figure 2**.

**Figure 2:**
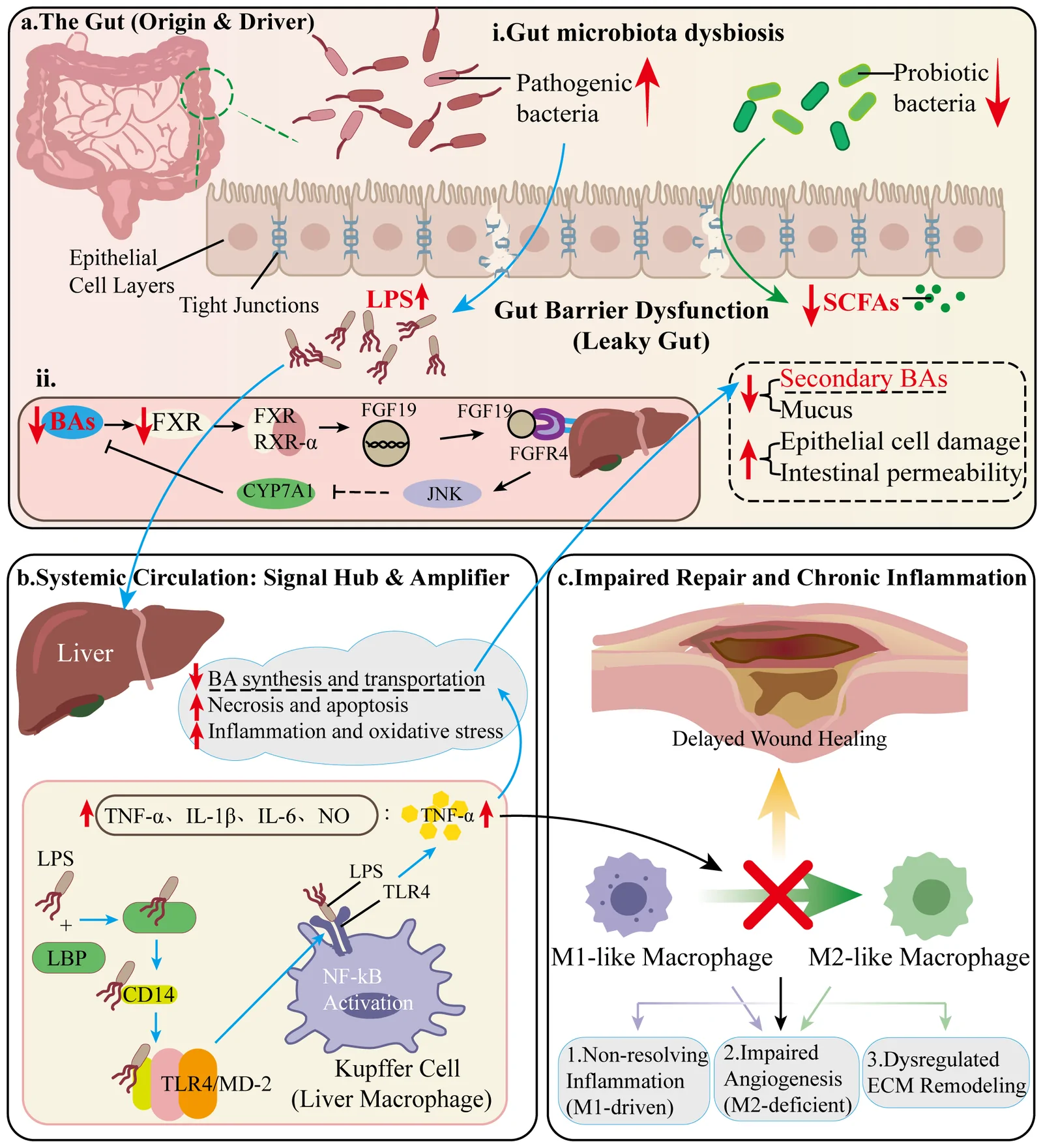
Schematic representation of signaling pathways along the gut–liver axis. Solid arrows denote pathways supported by experimental evidence; dashed arrows denote proposed links that have not yet been experimentally confirmed. **(a)** The gut: origin and driver. (i) Gut microbiota dysbiosis. In diabetes, the composition of the gut microbiota is altered (↑pathogenic bacteria, ↓probiotic bacteria), with ↓SCFAs and LPS↑ at the epithelial surface. Disruption of the epithelial cell layers and tight junctions results in gut barrier dysfunction (“leaky gut”). (ii) BA metabolism. Total BAs and FXR signaling are reduced (↓BAs, ↓FXR), and signaling through the FXR/RXR-α heterodimer to FGF19 and its receptor FGFR4, as well as CYP7A1 and JNK, is altered. In the mucus layer, secondary BAs are decreased (↓secondary BAs), with increased epithelial cell damage and increased intestinal permeability (↑epithelial cell damage, ↑intestinal permeability). **(b)** Systemic circulation: signal hub and amplifier. Gut-derived products reach the liver, where LPS is transferred by LBP to CD14 and then to the TLR4/MD-2 complex, activating NF-κB in Kupffer cells (liver macrophages). The liver shows decreased BA synthesis and transportation, increased necrosis and apoptosis, and increased inflammation and oxidative stress, with increased release of TNF-α, IL-1β, IL-6 and NO. **(c)** Impaired repair and chronic inflammation. These circulating mediators reach the wound, where the M1→M2 transition is blocked. This leads to non-resolving inflammation (M1-driven), impaired angiogenesis (M2-deficient) and dysregulated ECM remodeling, and ultimately to delayed wound healing. BA, bile acid; BAs, bile acids; CD14, cluster of differentiation 14; CYP7A1, cholesterol 7α-hydroxylase; ECM, extracellular matrix; FGF19, fibroblast growth factor 19; FGFR4, fibroblast growth factor receptor 4; FXR, farnesoid X receptor; IL-1β, interleukin-1β; IL-6, interleukin-6; JNK, c-Jun N-terminal kinase; LBP, lipopolysaccharide-binding protein; LPS, lipopolysaccharide; M1, M1-like (classically activated) macrophage; M2, M2-like (alternatively activated) macrophage; MD-2, myeloid differentiation factor 2; NF-κB, nuclear factor kappa B; NO, nitric oxide; RXR-α, retinoid X receptor alpha; SCFAs, short-chain fatty acids; TLR4, Toll-like receptor 4; TNF-α, tumor necrosis factor alpha.

First, gut microbiota dysbiosis and impaired barrier function (a). Previous studies have suggested that diabetes-related gut microbiota dysbiosis and its metabolites are elevated in patients with type 2 diabetes mellitus (T2DM) and are associated with poor wound healing (24). Based on this, we hypothesize that the production of local protective metabolites in the gut (such as SCFAs) may be reduced, while increased intestinal permeability (“leaky gut syndrome”) may allow bacterial products such as LPS to enter the liver via the portal venous system (6, 25). It should be noted that there are currently no direct studies validating this approach in the field of diabetic wounds.

Second, the potential role of enteric signals in the liver (b). The portal vein is the primary route through which enteric products enter the liver; within the liver, Kupffer cells can recognize LPS via toll-like receptor 4 (TLR4) and become activated, releasing TNF-α and IL-1β—this mechanism has currently been supported by experiments primarily in Metabolic Dysfunction-Associated Steatohepatitis (MASH) (26, 27). Based on this, we hypothesize that persistent exposure to endotoxins in the portal vein under diabetic conditions may keep Kupffer cells in an activated state and may contribute to disturbances in hepatic glucose and lipid metabolism, as well as the systemic release of TNF-α and IL-1β, via pathways such as PI3K/AKT/FOXO1 and mechanistic target of rapamycin complex 1 (mTORC1)/sterol regulatory element-binding protein 1c (SREBP-1c) (7, 8). However, it should be emphasized that the latter part of the statement is merely a reasonable extrapolation based on the underlying mechanisms: we have not identified any original studies that directly link “intestinal LPS–Kupffer cells” to the two metabolic pathways mentioned above and further associate them with wound healing; therefore, this aspect should be regarded as a hypothesis that requires further validation.

Finally, the potential role of macrophages as a bridge (c). As a bridge between the gut–liver axis and wound healing, macrophages may receive signals—such as LPS and inflammatory cytokines—from this axis, causing their surface receptors (such as TLR4) to be continuously activated. Consequently, by the time they reach the wound site, they may already have shifted toward a pro-inflammatory M1-like phenotype, while their responsiveness to healing signals is reduced (28, 29). Evidence supporting this hypothesis includes: TLR4/myeloid differentiation primary response 88 (MyD88)-dependent epigenetic reprogramming of macrophages in diabetic wounds promotes the M1-like phenotype and impairs healing (30); and myeloid cell and hematopoietic stem cell epigenetic conditioning driven by bacterial metabolites can transmit inflammatory and impaired-healing phenotypes to normal recipient mice via bone marrow transplantation (24). However, the two points—that “preprogramming signals originate from the liver” and that “macrophages are already programmed before reaching the wound site”—remain unproven by direct experimental evidence and constitute the core assumptions of this model.

#### 2.2.1 Intestinal dysbacteriosis and impairment of the intestinal barrier

##### 2.2.1.1 Intestinal dysbacteriosis

Gut dysbiosis, also referred to as intestinal microecological imbalance, is defined as the loss or gain of bacteria that promote health or disease, respectively, and in chronic metabolic disease it is characterized by reduced gut microbial diversity and altered abundance of specific taxa (31). In patients with DM, this dysbiosis is typically manifested as a reduction in beneficial commensal bacteria and an expansion of opportunistic pathogens (32). Gut dysbiosis is not only closely associated with the onset and progression of DM (32) but also exerts detrimental effects on diabetic wound healing (24, 33).

Gut dysbiosis not only reduces the production of SCFAs, thereby compromising intestinal barrier integrity, but may also increase the levels of certain bacterial metabolites, leading to an accumulation of pro-inflammatory metabolic products. This milieu readily triggers chronic low-grade systemic inflammation and further impairs insulin signaling, thereby exacerbating insulin resistance (7, 34). In diabetic wounds, such persistent inflammation interferes with normal healing, manifesting as macrophage dysfunction, impaired angiogenesis, and elevated levels of matrix metalloproteinases(MMPs).

##### 2.2.1.2 Impairment of intestinal barrier function

Gut dysbiosis can compromise intestinal barrier function, a phenomenon often referred to as “leaky gut”—and hyperglycemia itself disrupts epithelial tight junctions and increases permeability (35). As a result, intestinal permeability is increased, allowing bacterial endotoxins and their metabolites to more readily traverse the gut wall and enter the systemic circulation (7). For example, when intestinal permeability is elevated, LPS can more easily cross the intestinal wall into the portal and systemic circulation, thereby inducing metabolic endotoxemia (7); in humans, circulating markers of endotoxin exposure (lipopolysaccharidebinding protein (LBP), soluble CD14 (sCD14)) are elevated in obesity and are independently associated with metabolic and hepatic complications in adults with T2DM (36).

Damage to tight junction proteins is one important cause of intestinal barrier dysfunction. Tight junctions between intestinal epithelial cells are key structures that maintain barrier integrity and are primarily composed of protein families such as occludins and claudins. Sustained hyperglycemia and inflammation in diabetes can impair the expression and function of these tight junction proteins, thereby increasing intestinal permeability (35, 37).

At the same time, gut dysbiosis and leaky gut are considered major contributors to metabolic endotoxemia of intestinal origin. Persistent endotoxin exposure activates the TLR4 pathway in macrophages, promotes M1-like polarization, and enhances the release of pro-inflammatory cytokines, thereby further aggravating the inflammatory microenvironment in diabetic wounds (28).

#### 2.2.2 Hepatic metabolic dysfunction

The liver is a vital metabolic organ that plays a role in regulating the metabolism of glucose, lipids, and proteins. Impaired hepatic metabolic function can lead to systemic metabolic disorders (38), which may in turn reduce liver-derived circulating mediators required for wound repair, such as insulin-like growth factor-1 (IGF-1) (39). The liver receives blood directly from the intestines via the portal venous system, making it the first line of defense against intestinal toxins and microbial byproducts. Impaired intestinal barrier function—known as “leaky gut”—can cause bacterial byproducts and metabolites from the intestinal lumen to leak into the portal venous circulation and subsequently reach the liver (7, 35). As the first filter for blood entering the portal vein, the liver is responsible for clearing most endotoxins (26); however, under chronic high LPS exposure, the liver’s own immunometabolic balance is disrupted (26, 38). Kupffer cells in the liver recognize LPS via TLR4, activate the NF-κB and mitogen-activated protein kinase (MAPK) signaling pathways, and release pro-inflammatory cytokines such as TNF-α and interleukin-6 (IL-6) (26, 40). It is worth noting that the liver is not only the organ responsible for clearing LPS, but also acts as an amplifier of systemic inflammatory signals (29). When the liver is in a state of insulin resistance and lipid accumulation, its sensitivity to LPS is significantly increased, leading to the persistent presence of a low-grade chronic inflammatory state (26, 38). This liver-mediated inflammatory signal is not confined to liver tissue but spreads throughout the body via the bloodstream, providing a systemic inflammatory background that preprograms bone marrow immune cells (2, 3). Collectively, these two hepatic outputs—a diminished supply of liver-derived trophic mediators such as IGF-1 and an amplified systemic inflammatory tone—converge on the wound bed: reduced hepatic IGF-1 output delays re-epithelialization and impairs granulation tissue formation, collagen deposition, and neovascularization (39), whereas pro-inflammatory myeloid cells arising from a preprogrammed bone marrow compartment are sufficient to transfer the wound healing deficit to naive recipients (2). The liver, therefore, acts as a proximal determinant of wound repair capacity in diabetes, coupling gut-derived endotoxin exposure and hepatic metabolic dysfunction to impaired healing through both the loss of trophic support and the gain of systemic inflammatory drive (24).

The liver plays a pivotal role in glucose homeostasis, and dysregulation of hepatic glucose production and utilization contributes to the development of T2DM. Hepatic metabolic dysfunction implies that the liver becomes insensitive to insulin’s signal to suppress gluconeogenesis, while continuing to synthesize fat. This state is driven by impaired signaling through the PI3K/AKT/FOXO1 pathway and sustained or excessive activation of the mTORC1/SREBP-1c pathway (41). These alterations not only exacerbate hyperglycemia but may also compromise the energy metabolism and nutritional support required for proper wound healing.

The liver also produces IGF-1, which plays a crucial role in regulating cell growth, proliferation, and angiogenesis. In addition, dysregulation of hepatic phosphoenolpyruvate carboxykinase 1 (PCK1) is a key driver of abnormal hepatic gluconeogenesis in T2DM. Small interfering RNAs (siRNAs) targeting PCK1 can promote wound healing in T2DM-like mice by restoring glucose homeostasis (42).

The liver also plays an important role in immune regulation by secreting a wide range of cytokines and chemokines. Hepatic metabolic disturbances may alter the secretion profile of these immunomodulatory mediators, thereby indirectly affecting macrophage recruitment and polarization.

#### 2.2.3 Regulatory roles of gut–liver axis–derived metabolites

##### 2.2.3.1 Regulatory effects of short-chain fatty acids

SCFAs produced by gut microbial metabolism are regarded as key signaling molecules within the gut–liver axis (43). Among them, butyrate, propionate, and acetate are the principal SCFAs generated by bacterial fermentation of dietary fiber (44). In particular, butyrate is crucial for maintaining the energy supply of intestinal epithelial cells (45) and preserving barrier integrity. In patients with diabetes, gut dysbiosis frequently leads to reduced SCFA production (32), thereby compromising intestinal barrier function (35). SCFAs also exert anti-inflammatory effects through epigenetic mechanisms: by inhibiting histone deacetylases (HDACs), butyrate increases histone H3 acetylation at the Foxp3 promoter—the locus encoding the regulatory T cell (Treg) lineage–defining transcription factor—and thereby promotes the extrathymic generation and differentiation of Foxp3^+^ Tregs, fostering immune tolerance and suppressing inflammation (46, 47). In addition, SCFAs may modulate macrophage metabolism and polarization, promoting a shift toward an M2-like phenotype. It should be noted that Akkermansia muciniphila is not a canonical butyrate producer; it mainly produces propionate and acetate and may indirectly stimulate butyrate production through syntrophic interactions with butyrate-producing bacteria such as Faecalibacterium prausnitzii (44, 48).

##### 2.2.3.2 Regulatory roles of bile acids

BAs are hepatic metabolic products that are further transformed by the gut microbiota into secondary BAs in the intestine. As key components of the farnesoid X receptor (FXR)-fibroblast growth factor 19 (FGF19) axis, BAs act on nuclear receptors and membrane receptors, such as the farnesoid X receptor (FXR) and G-protein–coupled bile acid receptor 1 (TGR5), and play pivotal roles in regulating glucose and lipid metabolism, inflammatory responses, and intestinal barrier function (49). Alterations of gut dysbiosis in patients with diabetes may disturb the bile acid profile and its associated signaling pathways, thereby exacerbating hepatic metabolic dysfunction and systemic inflammation, and indirectly impairing wound healing. It should be noted that ursodeoxycholic acid (UDCA) has context-dependent effects on FXR: it has been reported to act as a weak indirect FXR antagonist by diluting the pool of more potent endogenous FXR agonists, while other studies suggest it may act as an ileal FXR agonist in certain settings (50). The claim that SCFAs directly enhance bile acid reabsorption requires further experimental support and is therefore presented here as a hypothesis rather than an established mechanism.

##### 2.2.3.3 Regulatory roles of LPS

LPS is a component of the outer membrane of Gram-negative bacteria, and its effects on innate immunity are strongly dose- and timing-dependent: low-dose or appropriately timed exposure can promote trained immunity (enhanced responsiveness to a secondary challenge), whereas sustained or high-dose exposure characteristically induces endotoxin tolerance (a refractory, hyporesponsive state) (51). It should be emphasized that “trained immunity” was originally defined in studies that used β-glucan and Bacillus Calmette-Guérin (BCG) as model stimuli rather than LPS (52). Nevertheless, in the metabolic context relevant here, LPS and other environmental factors such as free fatty acids signal through TLR4 and can establish a state of heightened secondary responsiveness that persists after the initial stimulus has subsided. This reprogramming involves epigenetic remodeling of bone marrow hematopoietic progenitor cells—including changes in activating histone marks such as H3K4me3 and H3K27ac at the promoters of pro-inflammatory genes in turn generates monocytes that display persistent morphological and functional changes, such as upregulation of the glycolytic enzyme system and suppression of mitochondrial function. Consequently, circulating monocytes already exhibit a “pro-inflammatory bias”—a preactivated state prone to polarization toward the M1-like phenotype—even before they reach the wound site. This preprogrammed state may explain why patients with diabetes show persistently elevated systemic inflammatory markers and impaired wound healing capacity even in the absence of obvious infection.

However, there is currently a lack of direct evidence indicating a dose-response relationship between circulating LPS concentrations and the M1-like/M2-like ratio of macrophages at the wound site; this mechanism still needs to be validated through a multilevel sampling study involving the portal vein, systemic circulation, and the wound site.

#### 2.2.4 Macrophages as a nexus between the gut–liver axis and wound repair

Macrophages are not only present at the wound site but are also widely distributed in the liver (as Kupffer cells) and in the intestine. Aberrant signaling along the gut–liver axis is associated with altered macrophage function in these tissues. For example, gut-derived LPS that reaches the liver activates Kupffer cells, provoking both local hepatic and systemic inflammation (53). Importantly, because Kupffer cells are tissue-resident macrophages that do not migrate to distant organs, the signals they transmit to the wound are humoral rather than cellular: their secreted cytokines and extracellular vesicles, together with other liver-derived circulating mediators, may be carried by the bloodstream to the wound site, where they may further promote M1-like polarization of wound-resident macrophages, thereby contributing to a vicious cycle (54). This constraint also applies to the intestinal arm of the axis: the mediators released by tissue-resident macrophage populations—not the cells themselves—are what reach the wound bed (**Figure 2**).

### 2.3 Gut–liver axis and epigenetic regulation of macrophages

Along the gut–liver axis, macrophages—particularly liver-resident Kupffer cells and intestine-derived monocytes—serve as critical immune sentinels whose functional phenotypes are substantially shaped by epigenetic regulation.

Gut microbiota dysbiosis is now recognized as a key driver in the pathogenesis of several liver diseases, including MASLD and MASH, as well as alcohol-associated liver disease, in which hepatic macrophages orchestrate liver inflammation, fibrosis and tissue repair.

Such reprogramming is enacted through DNA methylation, histone modifications and non-coding RNAs—epigenetic mechanisms that modulate gene expression without altering the underlying DNA sequence.

#### 2.3.1 DNA methylation

DNA methylation primarily occurs at the cytosine residues of CpG dinucleotide sequences and is typically associated with transcriptional silencing of genes. In diabetic wounds, an imbalance in DNA methylation and demethylation processes is considered a key factor contributing to macrophage dysfunction (55). Recent studies indicate that phenotypic conversion in macrophages involves both DNA methylation and active DNA demethylation, mediated respectively by DNA methyltransferase 1 (DNMT1) (2, 56, 57) and ten-eleven translocation 2 (TET2) (55). Particularly in the context of metabolic dysregulation (2, 3, 56) along the gut–liver axis (29), this bidirectional epigenetic regulatory mechanism is crucial for understanding the persistence of chronic inflammation and impaired tissue repair (56, 57).

As a maintenance methyltransferase, DNMT1 plays a central role in maintaining genomic methylation patterns and suppressing the expression of specific genes. In an LPS-induced inflammatory environment, activation of the TLR4/NF-κB signaling pathway does indeed lead to upregulation of DNMT1 expression, which in turn hinders the conversion of macrophages to the M2-like reparative phenotype by increasing the methylation levels of CpG islands in the promoter regions of anti-inflammatory genes (58). For example, high expression of DNMT1 suppresses autophagy-related genes through methylation modifications, thereby impairing the ability of macrophages to undergo M2-like polarization in Western diet-induced nonalcoholic fatty liver disease (56). In addition, DNMT1 influences macrophage motility by regulating the cell cycle and cholesterol accumulation; its absence or inhibition improves the recruitment of macrophages to wound sites and accelerates healing (57). In diabetic conditions, the N6-methyladenosine (m6A) methyltransferase Wilms tumor 1-associated protein (WTAP) further exacerbates impaired wound healing by epigenetically activating DNMT1 (59). This evidence suggests that DNMT1-mediated hypermethylation is indeed one of the key mechanisms leading to “inflammatory lock-in” in macrophages.

However, focusing solely on the inhibitory role of DNMT1 overlooks the contribution of active demethylation to the initiation of repair (55). TET2, an α-ketoglutarate-dependent dioxygenase, oxidizes 5-methylcytosine to 5-hydroxymethylcytosine, thereby initiating DNA demethylation (60), and has been implicated in diabetic wound repair (55). In diabetic wounds, TET2 is recruited to the promoter regions of genes such as matrix metalloproteinase-9 (MMP-9) through interaction with a long non-coding RNA (lncRNA) (TETILA). The consequences of this pathway depend on the magnitude, duration, and spatial distribution of MMP-9 activity. Transient and locally regulated MMP-9 activity may support extracellular matrix turnover during repair, whereas sustained or excessive activity can cause excessive matrix degradation and impede healing in chronic diabetic wounds. Thus, TETILA–TET2-mediated MMP-9 upregulation should not be interpreted as universally beneficial; any reparative effect would depend on appropriately timed and localized proteolysis balanced by endogenous protease inhibitors (55). TET2 is not only responsible for DNA demethylation but also serves as a key hub for integrating metabolic signals and immune responses; its function is easily compromised under hyperglycemic conditions, leading to dysregulation of the interferon response in endothelial and immune cells (61). Accordingly, TET2-mediated demethylation may facilitate the transition from persistent inflammation to repair when it supports coordinated expression of reparative genes, including M2-like programs, alongside controlled matrix remodeling (62). This proposed benefit cannot be inferred from increased MMP-9 transcription alone and requires consideration of wound stage, cellular context, and the balance between matrix degradation and deposition.

In summary, impaired wound healing in diabetes is not caused solely by methylation-mediated silencing via DNMT1, but rather results from an imbalance between methylation maintenance mediated by DNMT1 and demethylation activation mediated by TET2. Future therapeutic strategies should focus on restoring this epigenetic dynamic balance: on the one hand, by inhibiting DNMT1 or WTAP to lift the methylation-mediated suppression of anti-inflammatory genes (57, 59), on the other hand, active demethylation can be promoted by enhancing TET2 activity or supplying necessary metabolic cofactors (such as α-ketoglutarate). Combined with regulation of the gut-liver axis, intervening in epigenetic modifications using probiotics or metabolites (such as butyrate) will provide a more systematic therapeutic approach for hard-to-heal diabetic wounds (63).

#### 2.3.2 Histone modifications

Histone modifications, such as acetylation, methylation, and phosphorylation, regulate gene expression by altering chromatin structure.

Histone acetylation is typically associated with gene activation. The activity of HDACs can be inhibited by gut microbial metabolites such as butyrate, thereby promoting histone acetylation and activating anti-inflammatory genes (43). However, HDAC inhibition may be attenuated in diabetic conditions, leading to reduced levels of activating histone modifications such as H3K27ac and H3K4me3, which in turn suppress the transcription of genes associated with M2-like macrophages (such as Arg1, Il10, and Cd206) (64).

Histone methylation modifications exhibit a high degree of site specificity and functional complexity in the regulation of gene transcription; among these, H3K4me3 typically acts as an activating mark that promotes gene expression, while histone H3 lysine 27 trimethylation (H3K27me3) primarily exerts a repressive effect. Among the pathological mechanisms underlying impaired wound healing in diabetes, the expression of the macrophage histone demethylase jumonji domain-containing protein 3 (JMJD3) is significantly upregulated; by specifically removing the repressive modification H3K27me3, it lifts the epigenetic silencing of inflammatory genes (30). Further research has revealed that JMJD3 not only directly regulates chromatin states but also functionally interacts with the cyclic GMP-AMP synthase-stimulator of interferon genes (cGAS-STING) signaling pathway, synergistically amplifying the inflammatory response and causing the wound to remain in a state of chronic inflammation (65). At the same time, the H3K4me3 axis mediated by the histone methyltransferase mixed-lineage leukemia 1 (MLL1) has also been shown to be closely associated with macrophage-mediated inflammatory responses; targeted regulation of this axis helps restore macrophage plasticity, thereby promoting the healing of diabetic wounds (66). In addition, butyrate—a metabolite derived from the gut—broadly inhibits the activity of HDACs, including HDAC1, HDAC2, and HDAC3 (43, 67), leading to a global increase in histone acetylation (68); however, region-specific histone deacetylation at transcription start sites, accompanied by downregulation of the associated genes, has also been reported (69). This comprehensive remodeling of the epigenetic landscape does not rely on a single, unvalidated specific binding site, but rather dynamically and finely regulates the transcriptional dynamics of multiple inflammatory genes—including Ptgs2 [which encodes cyclooxygenase-2 (COX-2)]—by altering chromatin accessibility, thereby modulating the inflammatory phenotype of macrophages at the macrolevel (65).

Regarding histone lactylation, recent evidence indicates that lactate-derived lactylation regulates gene expression and has been linked to macrophage functional polarization. Zhang et al. demonstrated that histone lactylation is induced during the late phase of M1-like macrophage activation and promotes the expression of homeostatic genes, including Arg1, thereby facilitating a transition toward an M2-like reparative phenotype (70). Therefore, the notion that lactylation simply “locks” macrophages into M1-like contradicts the original literature. Instead, lactylation appears to play a context-dependent role in promoting the resolution of inflammation and tissue repair.

Therefore, understanding the interactions among these modifications and their synergistic regulation of key inflammatory pathways (such as STING) and genes (such as Ptgs2) is of great significance for developing epigenetic therapeutic strategies for diabetic hard-to-heal wounds.

#### 2.3.3 Regulation by non-coding RNAs

ncRNA, particularly microRNAs (miRNAs) and long non-coding RNAs (lncRNAs), play pivotal roles in the regulation of gene expression. They modulate gene expression primarily by promoting mRNA degradation or inhibiting mRNA translation. During diabetic wound healing, miRNAs have been shown to participate in the regulation of macrophage polarization. For example, mmu-miR-145a-5p promotes macrophage polarization toward the M2-like phenotype and thereby accelerates diabetic wound healing (71), whereas miR-21-5p facilitates the healing process by modulating the TGF-β1/SMAD7/TIMP3 signaling pathway (72). Moreover, exosome-mediated regulation of macrophage polarization is also critical for diabetic wound repair (73): exosomes derived from lean adipose tissue macrophages can deliver miRNAs that improve healing outcomes in diabetic wounds (74).

### 2.4 Metabolic checkpoints along the liver-intestine axis

The mechanism by which the gut-liver axis may contribute to diabetic wound healing through the immunometabolic reprogramming of macrophages is thought to involve the direct intervention of gut microbiota-derived metabolites in macrophage energy metabolism pathways. LPS, SCFAs, and BAs, as key signaling molecules, are associated with the inflammatory phenotype and tissue repair potential of macrophages, acting through checkpoints in the tricarboxylic acid (TCA) cycle and oxidative phosphorylation (OXPHOS).

Under pro-inflammatory conditions, large amounts of LPS endotoxin enter the portal venous circulation, activating TLR4 on the surface of liver Kupffer cells and monocyte-derived macrophages. This activation triggers the classic Warburg effect, namely a shift from highly efficient mitochondrial OXPHOS to aerobic glycolysis (40, 75). This process is driven by a specific disruption in the TCA cycle: LPS stimulation leads to the inhibition of succinate dehydrogenase (SDH) activity, resulting in the accumulation of the intermediate metabolite succinate. This, in turn, upregulates the expression of glycolytic enzymes such as hexokinase 2 by inhibiting prolyl hydroxylase to stabilize hypoxia-inducible factor-1α (HIF-1α), thereby locking in the M1-like pro-inflammatory phenotype (76, 77). At the same time, LPS induces the expression of immune-responsive gene 1 [IRG1, also known as aconitate decarboxylase 1 (ACOD1)], which catalyzes the conversion of cis-aconitate to itaconate. Itaconate acts as a competitive inhibitor of SDH and an activator of the nuclear factor erythroid 2-related factor 2 (NRF2) pathway, thereby exerting negative feedback regulation on excessive inflammatory responses (78, 79). However, in the hyperglycemic environment of diabetes, this protective metabolic reprogramming is often impaired, leading to chronic local inflammation at the wound site and impaired healing (79, 80).

Conversely, SCFAs produced by the fermentation of beneficial gut bacteria promote the conversion of macrophages to an OXPHOS-dependent M2-like reparative phenotype via G protein-coupled receptor 41/43 (GPR41/43, also known as free fatty acid receptor 3/2 (FFAR3/FFAR2)) receptors or by inhibiting HDACs. SCFAs activate the AMP-activated protein kinase (AMPK) pathway, enhancing mitochondrial biogenesis and fatty acid oxidation, thereby providing an energy foundation for anti-inflammatory functions and improving the host’s overall metabolic phenotype.

In addition, as the hub of the gut–liver axis, the liver’s Kupffer cells undergo “immune training” as metabolic flux passes through the portal vein. BAs fine-tune macrophage lipid metabolism and NOD-like receptor family pyrin domain containing 3 (NLRP3) inflammasome activity through FXR and TGR5 signaling (81); Downregulation of methyltransferase-like 14 (METTL14) in the liver drives pro-inflammatory polarization of monocyte-derived macrophages via the MyD88/NF-κB pathway, exacerbating the systemic inflammatory environment (82).

In summary, metabolites from the gut-liver axis are associated with the healing process of diabetic wounds, in part through their proposed modulation of the “metabolic switch” of macrophages—LPS-induced glycolysis is associated with sustained inflammation, while SCFAs and bile acids may promote OXPHOS and thereby contribute to repair. Targeting these metabolic checkpoints—such as supplementing SCFA precursors or modulating the bile acid pool—holds promise for breaking the metabolic lock-in of chronic inflammation and reactivating tissue regeneration processes.

## 3 Key knowledge gaps and challenges

### 3.1 Mechanisms by which gut-derived immunometabolites reprogram wound-resident macrophages

Previous studies have shown that molecules such as SCFAs produced by gut microbiota metabolism have systemic anti-inflammatory effects, and their production decreases in diabetic conditions. Currently, there is a relatively solid research foundation regarding the role of gut microbiota-derived SCFAs in the regulation of immune and metabolic processes. Existing research reveals that the regulation of the inflammatory phenotype of macrophages by butyrate is not a simple linear promotional relationship, but rather exhibits a significant concentration-dependent biphasic pattern. A recent study by Jiang et al. (83) provides direct evidence for such a biphasic pattern in human monocyte-derived macrophages: low butyrate concentrations (0.1 mM) suppress LPS-induced TNF-α production through a peroxisome proliferator-activated receptor gamma (PPAR-γ)-dependent mechanism, whereas high concentrations (10 mM) abolish this anti-inflammatory effect, induce IL-1β, reduce the anti-inflammatory cytokine IL-10, and promote macrophage cell death. At the same time, in the pathological microenvironment of diabetic wounds, macrophages are often exposed to butyrate concentrations below the effective threshold due to reduced butyrate bioavailability resulting from local hyperglycemia, oxidative stress, and gut microbiota dysbiosis. This “subthreshold” exposure not only fails to activate anti-inflammatory programs but may, on the contrary, maintain or exacerbate the M1-like pro-inflammatory state due to disruption of signaling pathways. Therefore, a major gap in current research lies in the lack of quantitative analysis of the precise local concentration distribution of butyrate in diabetic wounds and its dynamic relationship with the metabolic state of macrophages. Existing treatment strategies often overlook this concentration-threshold effect, resulting in the failure to achieve the pharmacological concentrations required to induce M2-like polarization when butyrate is applied topically, thereby limiting its therapeutic efficacy.

In addition to the classic HDAC inhibition mechanism, butyrate—as an endogenous ligand for G protein-coupled receptor 109A (GPR109A, also known as hydroxycarboxylic acid receptor 2 (HCAR2))—triggers downstream signaling pathways by activating this receptor, representing another key yet often underestimated mechanism that promotes the polarization of macrophages toward the M2-like phenotype (84, 85). Activation of GPR109A induces activation of the AMPK/peroxisome proliferator-activated receptor gamma coactivator 1-alpha (PGC-1α) signaling pathway, which in turn optimizes mitochondrial function and promotes OXPHOS—a metabolic hallmark of M2-like macrophages (84). In diabetic wound healing, GPR109A-mediated signaling helps inhibit the NF-κB pathway, reduce the secretion of pro-inflammatory cytokines such as TNF-α and IL-6, and simultaneously increase the expression of the anti-inflammatory cytokine IL-10 (84). However, most current studies on the mechanisms of action of butyrate in wound healing have focused on its epigenetic regulatory effects (43, 64, 68, 69), while neglecting the GPR109A-mediated metabolic reprogramming pathway. In fact, there is a potential synergistic or competitive relationship between GPR109A-mediated signaling and HDAC inhibition (86). Particularly in a hyperglycemic environment, mitochondrial dysfunction in macrophages may limit the progression of OXPHOS; in such cases, even if HDAC is inhibited, macrophages may struggle to complete their full conversion to the M2-like phenotype if the GPR109A pathway is blocked. Therefore, elucidating the interactive network between the GPR109A and HDAC pathways in diabetic macrophages represents a significant gap in current mechanistic research.

At the same time, there is currently a lack of direct research evidence addressing how metabolites in the circulation specifically target wound-site macrophages rather than systemic immune cells. Although there is preliminary evidence supporting the “gut-brain axis” and “gut-lung axis,” related research has not yet extended to the “gut-wound axis.” Furthermore, the genome-wide profile of modifications such as H3K27ac induced by HDAC inhibition in wound macrophages remains unclear (64). Evidence of p65 acetylation has been found in liver macrophages associated with MASH (87); wound-specific data are still largely lacking. Regarding the precise timing of the switch from glycolysis to OXPHOS on the wound repair timeline, it is known that the M1-like → M2-like transition is a key checkpoint; however, data on the temporal resolution of this metabolic switch remain insufficient. The direct causal relationship between reduced intestinal SCFA production in diabetic conditions and impaired wound healing has not yet been established, and current understanding is primarily based on inferences from associations.

Future advances in spatial metabolomics, single-cell omics, and coupled analysis of pattern recognition receptors and immunometabolic reprogramming may address these evidence gaps in the coming years (88, 89). Spatial analysis of tissue-specific macrophage immunometabolism will enable the characterization of macrophage metabolic niches in wound tissue and simultaneously resolve the transcriptomic, epigenomic, and metabolomic states of wound-resident macrophages. Spatial metabolomic profiling of SCFAs, BAs, and LPS in diabetic wound tissue should be prioritized to definitively establish whether gut–liver axis-derived metabolites actually reach the wound microenvironment.

### 3.2 Studies on vesicle-mediated priming of macrophages toward a pro-inflammatory state along the gut–liver axis

Current evidence indicates that hepatic Kupffer cells (54) and intestinal immune cells (90) both secrete exosomes that carry bioactive cargo, including proteins and miRNAs. Disruption of the gut–liver axis under diabetic conditions may alter the secretion profiles of these exosomes. For example, it has been shown that exosomes released from damaged liver tissue can transfer fatty acid synthase (Fasn) and thereby exacerbate alveolar bone loss in patients with T2DM (54). The action of administered exosomes and EVs on the diabetic wound bed can be organized along four variables: donor cell source, cargo, recipient cell type, and signaling pathway. Mesenchymal stromal cell–derived EVs shift wound macrophages toward an M2-like phenotype via PI3K/Akt, NF-κB, MAPK, and HIF-1α/vascular endothelial growth factor (VEGF) signaling, with increased CD31^+^ vessel density, granulation tissue, and collagen deposition in diabetic animals (73, 91); M2-like macrophage–derived vesicles act in an autocrine/paracrine manner on activated M1-like macrophages and reprogram their mitochondrial metabolism (92, 93). Evidence strength nevertheless differs: most studies demonstrate an association between EV administration and improved healing with M2-like marker expression (73, 91–93), rather than the necessity and sufficiency of a defined cargo in a defined recipient cell—although a few studies have defined both the cargo and the recipient cell (94)—and effect sizes are not comparable across donor cells, isolation methods, and dose units.

Conditional Cre drivers (Rab27a^fl/fl^ × LysM-Cre) are intended to block macrophage exosome secretion but do not trace vesicles (95), and Rab27a also regulates secretory lysosomes and exosome-independent secretion (96), so loss of function is not exosome-specific and must be validated by quantifying secreted vesicles (97); either way, the loss-of-function side must be paired with an explicit labeling strategy (95, 96). Lipophilic dyes (DiR, PKH26) transfer to neighboring cells and to co-isolated lipoproteins even without vesicle uptake, and self-assemble into micelles indistinguishable from labeled vesicles by scatter and fluorescence; fewer than 5% of vesicles colocalize with such dyes (98–101). Biodistribution must therefore be separated from genuine uptake by dye-only and dye-plus-vehicle controls. Genetically encoded reporters avoid these artifacts but have their own limits: CD63-emGFP and inducible CD63-NanoLuc mice allow cell-type-specific *in vivo* tracking (102, 103), yet CD63 and CD9 label partly distinct populations—exosomes versus ectosomes (104) — and CD63-NanoLuc tagging itself shifts biodistribution toward the lungs (105). Assigning recipient cell identity further requires co-staining EV reporter signal with macrophage markers (F4/80, CD68, CD206) on tissue sections, as achieved with ^89^Zr-labeled EVs that colocalized with CD206^+^, F4/80^+^, and IBA1^+^ subsets (106).

Standardization is a further limitation, and most wound-healing EV studies do not follow Extracellular Vesicles (MISEV) consensus reporting (107). Isolation method changes the preparation—ultracentrifugation co-isolates lipoproteins and protein aggregates, whereas size-exclusion chromatography and tangential flow filtration yield different particle-to-protein ratios and lipoprotein content sufficient to alter measured uptake in the same system (98, 101). Dose is reported variously as particle number, protein mass, or cell equivalent; potency assays, storage stability, and batch-to-batch variability are seldom quantified, so efficacy claims are not comparable across studies. Future wound-healing studies should report donor cell source and passage, isolation method, particle and marker characterization, dose unit, route and schedule, storage conditions, and a defined potency readout, and should adopt existing consensus guidance rather than defining study-specific criteria (107). These factors, rather than biological potency alone, currently limit clinical translation.

Direct evidence in the diabetic wound setting remains therapeutic rather than mechanistic: in diabetic rats, human umbilical cord MSC–derived exosomes reduced wound area with increased CD206, CD31 and VEGF expression and greater collagen deposition (73), and in diabetic mice, adipose-derived MSC exosomes delivered on a human acellular amniotic membrane accelerated healing by regulating inflammation and stimulating vascularization (91). By contrast, mechanistic evidence has so far been obtained only outside the wound setting: in type 2 diabetic mice, exosomes from metabolically impaired liver were enriched in the periodontal region and aggravated alveolar bone loss through transfer of Fasn (54). No study has yet traced endogenously released gut- or liver-derived exosomes into the diabetic wound bed, identified the recipient macrophage subset, or shown that the transferred cargo is necessary for the M1-like bias.

Although existing studies provide clues that the gut–liver axis may influence diabetic wound healing via exosomes, there is currently no direct evidence that circulating exosomes of gut–liver axis origin exhibit characteristic alterations in their miRNA, protein, or lipid signatures under diabetic conditions, nor is it known whether such changes can mediate epigenetic or metabolic pre-programming of bone marrow progenitors or monocytes before they reach the wound, thereby intrinsically biasing them toward pro-inflammatory M1-like macrophage polarization.

At the same time, data remain scarce regarding the relative contributions of liver-derived versus gut-derived exosomes to the diabetic wound microenvironment, as well as the spatiotemporal dynamics of exosome uptake *in vivo*. Moreover, causal evidence that exosomes regulate the fate decisions of macrophage precursors is still lacking and will require validation through multi-organ tracing, conditional disruption of exosome secretion with blockade verified by quantifying secreted vesicles and paired with a matched gain-of-function labeling strategy (e.g., Rab27a^fl/fl^ × LysM-Cre or Vil1-Cre mouse models), and integrated single-cell multi-omics analyses.

To elucidate this form of “systemic pre-programming”, future studies could combine exosome lineage tracing, single-cell assay for transposase-accessible chromatin with sequencing (scATAC-seq)-plus-RNA sequencing of diabetic wound tissue, and *in vitro* co-culture systems using human organoids and monocytes, with mandatory dye and reporter artifact controls and macrophage–reporter co-localization to assign recipient identity (99, 100, 106) and MISEV-based reporting (107). These approaches will help to dissect the origins of inflammatory dysregulation in diabetic wounds and provide a theoretical foundation for the development of novel therapeutic strategies.

### 3.3 Knowledge gaps in the mechanisms by which gut–liver axis dysfunction–induced systemic inflammation breaches local barriers and exacerbates immune imbalance in wounds

In diabetes, gut microbiota dysbiosis and impaired intestinal barrier function are common, allowing bacterial endotoxins such as LPS to cross the intestinal wall and enter the circulation (108). As a potent pro-inflammatory molecule, LPS activates immune cells throughout the body and drives the release of large amounts of pro-inflammatory cytokines. However, the mechanisms underlying the selective extravasation and accumulation of systemic inflammatory mediators such as IL-1β, TNF-α, and S100A8/A9 (109) within the microvasculature of diabetic wounds have not yet been fully elucidated. Furthermore, how these systemic signals act in concert with local factors such as hyperglycemia and AGEs to establish an apparently “irreversible” immune microenvironment that suppresses M2-like polarization and favors persistent M1-like retention remains a major challenge for future research. It is essential to identify the key interfaces and nodes at which systemic signals and local factors intersect and communicate.

### 3.4 Spatiotemporal coordination and individualized optimization of multi-target interventions along the gut–liver axis

It is now recognized that single interventions such as FMT, probiotics, SCFA supplementation, and bile acid receptor agonists show certain therapeutic effects in animal models, although the endpoints assessed differ across modalities: FMT and probiotics promote tissue regeneration in type 2 diabetic mice (110); oral SCFA (butyrate) supplementation reduces systemic inflammation and improves gut barrier integrity in db/db mice (111); and bile acid receptor agonists modulate hepatic inflammation and monocyte/macrophage phenotype in obese diabetic mice (112). However, whether synergistic or antagonistic interactions exist among these interventions, and how to achieve precise spatiotemporal coordination and individualized optimization, remain key unresolved questions.

Research on the regulatory mechanisms of the gut-liver axis in diabetic wound healing is currently facing a critical bottleneck in the transition from descriptive correlations to causal validation. Although existing literature has widely confirmed a significant association between gut microbiota dysbiosis, elevated levels of systemic inflammatory mediators, and delayed wound healing in diabetes, there remains a lack of conclusive experimental evidence to establish a direct causal chain among the distant organs—the gut, liver, and skin (110). Given the lack of validation of the “gut-liver axis–macrophage–wound” causal chain, future research strategies must move beyond traditional observational analyses and shift toward in-depth mechanistic analyses using conditional knockout models (such as LysM-Cre-mediated macrophage-specific TLR4 knockout or hepatocyte-specific FXR knockout) in combination with wound healing models and FMT, in order to bridge the current knowledge gap between systemic preprogramming and targeted therapies.

In addition, future research could explore the potential synergistic effects of supplementing with SCFAs and modulating bile acid signaling pathways. For example, SCFAs may influence bile acid metabolism by regulating the gut microbiota (113). Bile acid receptor agonists, such as obeticholic acid (OCA), can regulate the composition and metabolic function of the gut microbiota by activating FXR (49, 114).

To achieve precise spatiotemporal release of therapeutics along the “intestine–liver–wound” axis, smart stimuli-responsive nanocarrier technologies offer promising solutions (92, 115). Such systems need to respond to physiological cues characteristic of the wound microenvironment, including pH, enzymatic activity, or redox status. In parallel, genetically engineered probiotics can be designed to achieve targeted and sustained production and release of specific bioactive molecules, such as SCFAs and anti-inflammatory cytokines. Encapsulating engineered probiotics within smart carriers and delivering them via nanodelivery systems can both protect their viability in the gastrointestinal tract and enable precise release in the intestine, thereby modulating the gut microecology and the gut–liver axis (116, 117). These two technological avenues may be combined in future work to design highly precise drug-release strategies.

Most current intervention studies lack precise control strategies; achieving individualized optimization will require integrating multi-omics datasets, including metagenomics, metabolomics (32), host genotypes, and single-cell transcriptional profiles of wound-resident macrophages. On the basis of these datasets, constructing “digital twin” models capable of predicting treatment responses and guiding dynamic adjustment of therapeutic regimens may ultimately enable truly precise and individualized management of diabetic wound healing.

### 3.5 Challenges in clinical translation

Despite substantial progress in elucidating the impact of the gut–liver axis and macrophages on diabetic wound healing, critical knowledge gaps remain. In particular, significant challenges persist in implementing personalized therapeutic strategies based on the gut–liver axis–immune network. Impaired healing of diabetic wounds represents a complex pathophysiological process encompassing multiple dimensions, including chronic hyperglycemia, local hypoxia, oxidative stress, dysregulated inflammation, and cellular dysfunction (118). Furthermore, patients exhibit substantial heterogeneity in gut microbiota composition, metabolic phenotypes, and immune responses. Recent studies have underscored the pivotal role of the gut microbiota in this context; notably, gut microbiota dysbiosis has been demonstrated to be closely associated with the pathogenesis and progression of diabetes and its complications (including impaired diabetic wound healing) (24, 33). However, feasibility studies regarding personalized therapies based on the gut–liver axis–immune network are scarce, and specific methodologies to predict therapeutic responses to FMT, specific probiotics, or metabolite supplementation based on patients’ baseline microbiota characteristics and metabolic profiles—crucial for achieving true personalized precision medicine—are indeed absent. This represents one of the most significant challenges facing clinical translation.

Achieving clinical translation still requires overcoming the following challenges. First, integrating multi-omics data to precisely identify “gut–liver–immune” endotypes associated with impaired diabetic wound healing is essential (119). This necessitates rigorous experimental designs incorporating gnotobiotic mouse colonization assays, bone marrow chimera models, and liver-specific gene knockout models to establish a definitive causal link among the gut–liver axis, macrophages, and wound healing (24, 39).

Furthermore, establishing baseline biomarkers presents another significant challenge. To enable personalized treatment, it is essential to develop panels of baseline biomarkers capable of predicting patient responses to specific interventions (e.g., FMT, probiotics, or metabolite supplementation) (32). Such biomarkers should reflect the patient’s gut microbiota characteristics, metabolic profile, and immune status (25, 32).

In addition to the challenges outlined above, several other issues related to clinical translation should not be overlooked. First, inter-individual variability is a major concern: the pronounced heterogeneity of the microbiome across patients implies that standardized interventions may have limited efficacy. Therefore, it will be necessary to develop precision interventions tailored to individual patient profiles. In addition, the safety of interventions and the efficiency of drug delivery warrant careful consideration. The long-term safety of FMT remains to be fully established, particularly with respect to potential risks such as transfer of antibiotic resistance genes and excessive immune activation (120, 121). Rigorous donor screening and ethical oversight are therefore essential. Achieving efficient targeting and stable colonization of orally administered probiotics or metabolites in the gut also remains challenging. Emerging delivery technologies, such as microneedle-based systems, represent an important therapeutic direction and may offer novel strategies for localized treatment of wounds (63).

In parallel, clinical trials should incorporate standardized endotype-based stratification. Current clinical trial designs often fail to adequately account for differences in patients’ “gut–liver–immune” endotypes, resulting in heterogeneous therapeutic responses (120, 121). Future studies should conduct prospective, multicenter, biomarker-guided, stepwise intervention trials to validate the efficacy of personalized therapeutic strategies.

## 4 Clinical translation priorities

The central pathological feature of impaired diabetic wound healing is the failure of macrophages to transition from a pro-inflammatory M1-like phenotype to a pro-repair M2-like phenotype, which results in wounds being trapped in a state of persistent inflammation. The hyperglycemic microenvironment “locks” macrophages into a pro-inflammatory state by inducing metabolic reprogramming and aberrant epigenetic modifications, whereas the gut–liver axis—including bile acid metabolism and gut microbiota-derived metabolites—plays a pivotal role in regulating systemic immunometabolic homeostasis. The principal mechanisms by which hyperglycemia and gut–liver axis dysfunction converge on macrophage polarization are summarized in **Figure 3**.

**Figure 3:**
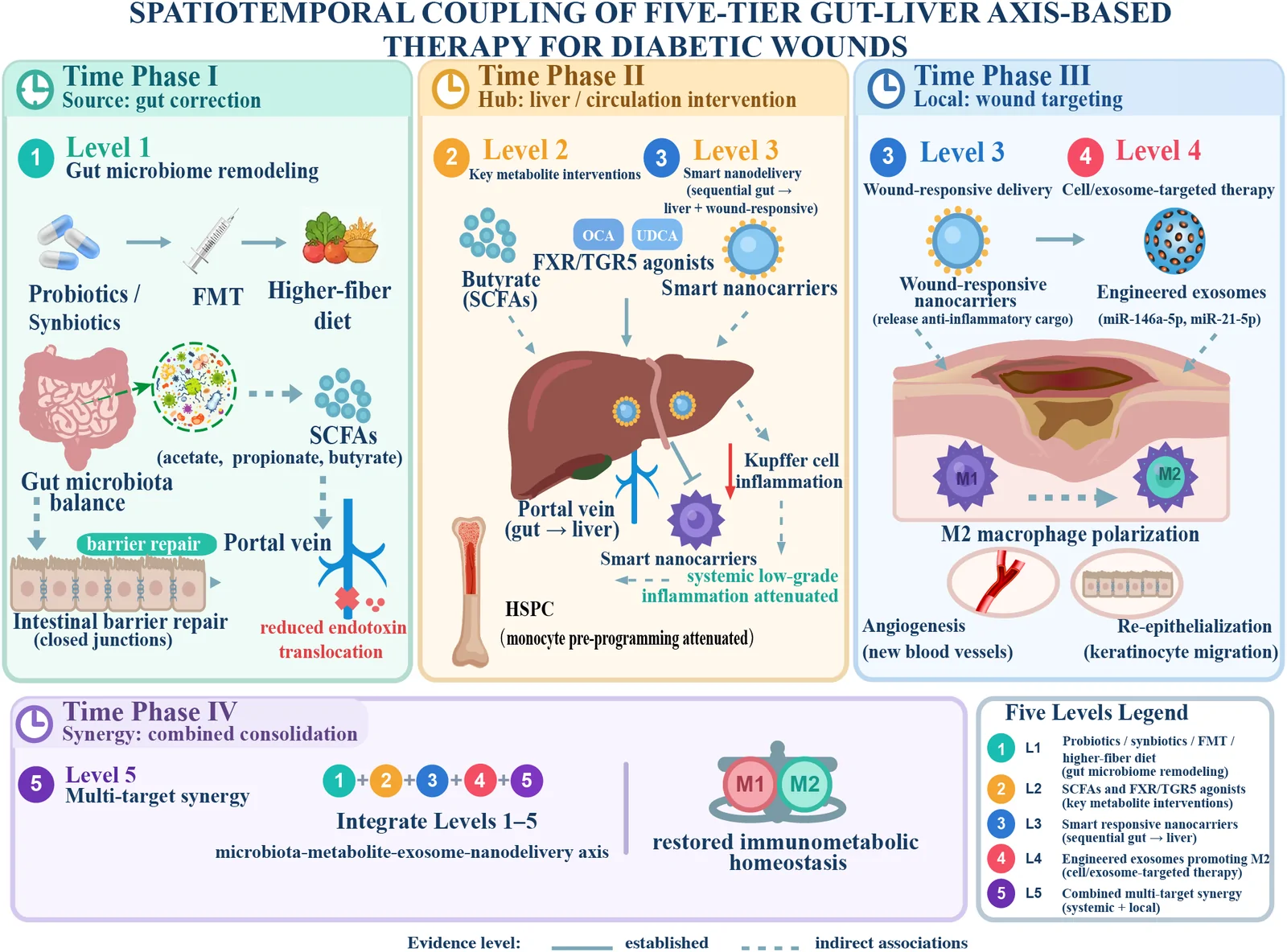
Schematic diagram of multi-level therapeutic strategies for diabetic wounds based on the gut–liver axis–macrophage regulatory network. The schematic illustrates a sequential, multi-level therapeutic strategy targeting the gut–liver–wound axis. Time Phase I (Source: gut correction) involves Level 1 (gut microbiome remodeling) through probiotics/synbiotics, fecal microbiota transplantation (FMT), and diet, leading to gut microbiota balance, intestinal barrier repair, and reduced endotoxin translocation. Time Phase II (Hub: liver/circulation intervention) comprises Level 2 (key metabolite interventions) using butyrate (short-chain fatty acids, SCFAs) and FXR/TGR5 agonists (OCA, UDCA), and Level 3 (smart nanodelivery) with sequential gut→liver plus wound-responsive smart nanocarriers. These interventions target the liver, attenuate Kupffer cell inflammation and systemic low-grade inflammation, and reverse monocyte pre-programming in hematopoietic stem and progenitor cells (HSPCs). Time Phase III (Local: wound targeting) involves Level 3 (wound-responsive delivery of anti-inflammatory cargo) and Level 4 (cell/exosome-targeted therapy with engineered exosomes carrying miR-146a-5p and miR-21-5p), promoting M2-like macrophage polarization, angiogenesis, and re-epithelialization. Time Phase IV (Synergy: combined consolidation) integrates Levels 1–5 (Level 5, multi-target synergy) through the microbiota–metabolite–exosome–nanodelivery axis to restore immunometabolic homeostasis (M1-like/M2-like balance). FMT, fecal microbiota transplantation; SCFAs, short-chain fatty acids; OCA, obeticholic acid; UDCA, ursodeoxycholic acid; FXR, farnesoid X receptor; TGR5, G protein–coupled bile acid receptor 1; HSPCs, hematopoietic stem and progenitor cells; miRNA, microRNA. M1, classically activated (pro-inflammatory) macrophage phenotype; M2, alternatively activated (pro-repair) macrophage phenotype.

Throughout this section, evidence is graded according to the framework used in **Table 1** and **Supplementary Table 1**: Preclinical = animal or *in vitro* studies; Early clinical = Phase 1/2 or small RCT; Clinical = Phase 3 or larger RCT; Hypothetical = no experimental validation. Importantly, the evidence level indicates proximity to clinical application, not certainty. For DFU-specific wound healing, no intervention has yet reached the “Clinical” level; the strongest wound-specific evidence is “Early clinical” from small RCTs. Therefore, statements below should be read as preliminary or hypothesis-generating unless explicitly labeled otherwise.

**Table 1:** Summary of proposed interventions targeting the gut–liver–macrophage axis in diabetic wound healing.

Axis level	Strategy category	Intervention	Primary target(s)	Evidence level & DFU status
Gut	Gut microbiota	Probiotics/synbiotics	Gut microbiota (113, 137); intestinal barrier (37)	Early clinical; small RCT in DFU (123)
Gut	Gut microbiota	FMT	Gut microbiota (120)	Preclinical for DFU (110); early clinical in T2D/obesity (mixed clinical results) (120, 121)
Gut	Gut metabolites	SCFA supplementation	Macrophage reprogramming (67, 83); gut barrier (151)	Preclinical (111); no DFU trials
Liver/bile acid	Liver/bile acid	OCA/FXR agonists	FXR/TGR5 signaling (112, 114)	Preclinical for DFU-specific study (114); Phase 3 in MASH (125, 126)
Liver/bile acid	Liver/bile acid	UDCA	Context-dependent FXR modulation (50)	Clinical in obesity, non-DFU (50); no targeted DFU trials
Systemic/wound	Systemic/wound macrophage	MSC-derived exosomes	Wound macrophages; angiogenesis	Early clinical; RCT (n = 110) completed for DFU (130)
Systemic/wound	Systemic/wound macrophage	Engineered exosomes (miRNA-loaded)	Wound macrophages (71); angiogenesis (4)	Preclinical (4, 71, 72); no DFU trials
Cross-cutting	Cross-cutting enabling technology	Smart responsive nano-delivery system	Wound bed (ROS-, MMP-9- and glucose-responsive delivery) (92, 93, 115)	Preclinical (132); no DFU RCTs

DFU, diabetic foot ulcer; FMT, fecal microbiota transplantation; FXR, farnesoid X receptor; MASH, metabolic dysfunction-associated steatohepatitis; miRNA, microRNA; MSC, mesenchymal stem cell; OCA, obeticholic acid; RCT, randomized controlled trial; SCFA, short-chain fatty acid; T2D, type 2 diabetes; TGR5, G protein–coupled bile acid receptor 1; UDCA, ursodeoxycholic acid. Evidence levels: Preclinical = animal or *in vitro* studies; Early clinical = Phase 1/2 or small RCT; Clinical = Phase 3 or larger RCT. A detailed version of this table, including study models, delivery routes, main findings, and references, is provided as **Supplementary Table 1**.

### 4.1 Microbiota-based interventions

The gut microbiota modulates systemic inflammatory tone and immune cell function through metabolites such as SCFAs and secondary BAs. In the context of diabetic wound repair, microbiota-based strategies mainly include: (i) administration of probiotics or synbiotics to restore intestinal microecological balance, increase the abundance of butyrate-producing bacteria (e.g., Faecalibacterium prausnitzii, Roseburia spp.); these approaches are hypothesized to alleviate wound-site inflammation via the “gut–skin axis”—the bidirectional crosstalk between the intestinal microbiota and the skin, in which gut-derived microbial metabolites and immune mediators shape cutaneous immune homeostasis and inflammation and cutaneous signals can in turn modify the gut microbial community (122). This crosstalk is supported primarily by preclinical models, and direct evidence that gut-derived metabolites reach the human wound bed is lacking. This strategy has reached early clinical testing in DFU (Early clinical; small RCT, **Table 1**): in a double-blind randomized controlled trial of patients with diabetic foot ulcers, a topical probiotic formulation in soybean concentrate significantly accelerated complete healing compared with placebo (mean 89.6 vs. 165.9 days, P = 0.012), and was safe and well tolerated (123). This finding is preliminary: it derives from a single small RCT, and the formulation is a soybean-concentrate topical product rather than a conventional probiotic. (ii) Modulation of the bile acid–FXR/TGR5 signaling axis: the gut microbiota can chemically modify BAs to augment or attenuate FGF19 signaling, thereby regulating hepatic lipid metabolism and systemic inflammation; targeting this axis may ameliorate metabolic dysregulation and indirectly improve the wound-healing microenvironment (124); the clinical safety profile of FXR-directed agents is discussed in the next section (125, 126). (iii) Fecal microbiota transplantation, as a more comprehensive intervention, which can globally reshape gut microbial communities and improve metabolic and immune functions (127). In a 24-week, double-blind, randomized controlled trial in obese patients with type 2 diabetes, repeated FMT significantly increased engraftment of lean-donor microbiota and the abundance of butyrate-producing bacteria, and reduced total and low-density lipoprotein (LDL) cholesterol and liver stiffness, without serious adverse events (120); in contrast, a double-blind placebo-controlled trial of weekly oral FMT capsules in adults with obesity and insulin resistance found gut microbiota engraftment but no significant improvement in insulin sensitivity (121), indicating that clinical benefit is not uniform. No randomized controlled trial has evaluated FMT for DFU wound healing; the cited RCTs assessed metabolic endpoints (insulin sensitivity, cholesterol, and liver stiffness) rather than wound closure, so evidence for FMT in DFU remains preclinical. FMT also carries risks of pathogen transmission and unpredictable long-term microbiome engraftment, which are particularly relevant in diabetic patients with chronic, often colonized wounds. The overarching rationale of these approaches is to restore intestinal barrier integrity, increase the production of anti-inflammatory metabolites (such as butyrate), and reduce endotoxemia, thereby correcting the systemic pro-inflammatory bias of macrophages and providing a rationale for investigating microbiota-targeted strategies in DFU.

### 4.2 Metabolite-based interventions

Disordered energy metabolism is a major intrinsic driver of diabetic foot ulcer development and progression; through oxidative stress and chronic inflammation, it induces metabolic reprogramming and functional imbalance in immune and reparative cells. Targeting this central process, metabolite-based supplementation aims to precisely correct key metabolic nodes: Butyrate salts, which act as histone deacetylase inhibitors, have been shown preclinically to restore suppressed histone acetylation in the diabetic microenvironment and reprogram the macrophage transcriptome to enhance phagocytic capacity and coordination of inflammatory responses (64); Bile acid receptor agonists (e.g., obeticholic acid) have been shown preclinically to activate FXR/TGR5, inhibit NF-κB signaling, reduce pro-inflammatory mediators, and promote macrophage M2-like polarization (112, 124); no DFU trials have tested this approach. Clinical data provide preliminary safety boundaries for this drug class: in the phase 3 REGENERATE trial, pruritus was the most common adverse event and was dose-dependent (19% with placebo vs. 28% with obeticholic acid 10 mg and 51% with 25 mg daily), while serious adverse events occurred at similar rates across groups (125); a subsequent analysis comprising more than 8,000 patient-years of exposure confirmed that pruritus remained the most common treatment-emergent event and that adjudicated hepatic, renal, and cardiovascular events were low and comparable across groups (126). These findings indicate that FXR agonism requires careful dose selection and monitoring before it can be considered for metabolic wound care. These REGENERATE safety data derive from a MASH population, not from patients with DFU. Extrapolating this tolerability profile to wound care is uncertain, particularly because DFU patients are typically older and have more comorbidities and polypharmacy. It should be noted that UDCA has context-dependent effects on FXR and is not a classical FXR agonist. With respect to lactate metabolism, hyperglycemia-driven enhancement of aerobic glycolysis leads to excessive lactate accumulation; while lactate-derived histone lactylation has been linked to the resolution of inflammation and M2-like polarization, the precise role of lactylation in diabetic wound macrophages requires further investigation (70). Furthermore, these four routes constitute the classical pathways through which hyperglycemia inflicts cellular damage: the polyol (aldose reductase) pathway, advanced glycation end-product (AGE) formation, protein kinase C (PKC) activation, and the hexosamine biosynthetic pathway. Notably, all four are activated downstream of excessive mitochondrial superoxide production—normalizing mitochondrial reactive oxygen species (ROS) prevents glucose-induced PKC activation, AGE formation, and sorbitol accumulation (128), and blocking mitochondrial superoxide overproduction with uncoupling protein-1 or manganese superoxide dismutase reverses the hyperglycemia-induced hexosamine-mediated changes in Sp1 O-GlcNAcylation (129). Addressing excessive mitochondrial superoxide as this shared upstream event is therefore therapeutically attractive: supplementation with antioxidant metabolites might attenuate damage mediated by the polyol pathway, AGE formation, PKC activation, and the hexosamine pathway. Collectively, these approaches represent a preclinical therapeutic direction for DFU centered on metabolic regulation. Notably, these mechanistic findings were established largely in non-wound models and have not been directly validated in diabetic wound tissue; therapeutic translation therefore remains preclinical.

### 4.3 Cell-based and exosome-based therapies

Stem cell–derived exosomes have emerged as a highly promising cell-free regenerative strategy for diabetic wound repair. Their key mechanism of action lies in the delivery of bioactive cargo—including miRNAs, proteins, lipids, and cytokines—which modulates critical signaling pathways such as PI3K/Akt, NF-κB, MAPK, TGF-β/Smad, and HIF-1α/VEGF. In turn, this promotes angiogenesis, accelerates fibroblast and keratinocyte proliferation, drives re-epithelialization, and restores immune balance by inducing M2-like macrophage polarization (118). Among the therapies discussed here, this has the most direct wound-specific clinical evidence to date, but it remains Early clinical (small RCT, n = 110): in a randomized controlled clinical trial of 110 patients with persistent diabetic foot ulcers, topical Wharton’s jelly mesenchymal stem cell–derived exosomes combined with standard of care significantly increased the proportion of patients achieving complete healing and shortened the mean time to complete epithelialization (approximately 6 weeks vs. 20 weeks in controls) (130). Exosome-based therapy is hypothesized to offer safety advantages by avoiding the risks of tumorigenicity and immune rejection associated with direct stem cell transplantation; however, no head-to-head clinical trial has compared these modalities in DFU, and this claim remains inferential. In addition to indirectly modulating immune cells via exosomes, directly targeting macrophages themselves (still preclinical; no DFU trials) (94)—for example, by manipulating their polarization state, metabolic phenotype, and spatiotemporal dynamics—represents another important therapeutic avenue (64, 131). Together, these strategies may provide a multidimensional therapeutic toolkit for the repair of chronic, non-healing diabetic wounds.

### 4.4 Nanodelivery systems

Nanodelivery systems represent a promising preclinical platform designed to achieve precise and localized delivery of therapeutic strategies, potentially enhancing the stability, residence time, and targeted release of exosomes and therapeutic molecules at the wound site. In the context of macrophage modulation, this platform is proposed to offer three major advantages: first, spatiotemporally controlled release, whereby specific bioactive molecules are released in a programmed sequence tailored to the requirements of distinct wound-healing phases (inflammatory, proliferative, and remodeling) (132); second, specific targeting of wound-resident macrophages via surface functionalization, which increases local drug concentrations while reducing systemic side effects; and third, support for multimodal combination therapy by integrating anti-inflammatory agents, pro-angiogenic factors, and extracellular matrix remodeling cues within a single delivery system to coordinately regulate macrophage phenotype and function (92, 132). Claims that such systems restore matrix architecture must, however, be verified histologically rather than inferred from macrophage phenotype alone: collagen density and the collagen I/III ratio by Masson trichrome or picrosirius red staining under polarized light, re-epithelialization and granulation tissue formation by hematoxylin and eosin (H&E) staining, and matrix balance by MMP-9/tissue inhibitor of metalloproteinases-1 (TIMP-1) immunohistochemistry (133, 134). It should be acknowledged, however, that this platform remains predominantly preclinical: to date, no randomized clinical trial has evaluated macrophage-targeted nanodelivery in diabetic foot ulcers, and the available clinical experience in this setting is limited to early-phase studies of advanced biologic delivery, such as the exosome trial described above (130). These features position nanodelivery systems as a conceptually enabling platform that could, in principle, support the staged delivery of therapeutics, although it remains exclusively preclinical for macrophage-targeted DFU applications (**Table 1**; detailed evidence in **Supplementary Table 1**).

Assessment of extracellular matrix remodeling and selection of experimental models. The matrix-related consequences of altered macrophage function require direct histological assessment rather than inference from cytokine or polarization data. Collagen density and the collagen I/III ratio should be quantified by Masson trichrome staining or, preferably, picrosirius red staining under polarized light, which distinguishes thin type III from thick type I fibers (134); re-epithelialization and granulation tissue formation should be assessed on H&E-stained sections; and matrix balance should be evaluated by MMP-9 and TIMP-1 immunohistochemistry, ideally expressed as the MMP-9/TIMP-1 ratio, since this ratio—rather than MMP-9 alone—predicts healing in diabetic wounds and increases in diabetic skin after injury (133, 135). This battery is particularly necessary for the proposed TET2–MMP-9 promoter demethylation mechanism discussed above, for which no collagen- or matrix-related readout has yet been reported: without such data, increased MMP-9 transcription cannot be linked to the matrix-degradation phenotype that impairs healing. The choice of animal model is an equally important limitation. For testing the gut–liver axis hypothesis, type 2 diabetes models—db/db mice or high-fat diet (HFD) plus low-dose streptozotocin (STZ)—are the appropriate choice, because they recapitulate the triad of gut dysbiosis, hepatic steatosis, and low-grade systemic inflammation that defines the proposed peripheral-to-core sequence (136, 137); STZ-induced type 1 diabetes models lack the obesity- and dysbiosis-driven hepatic phenotype and should therefore be used only as comparisons, not as the primary validation model. Finally, gut–liver axis causality cannot be established without microbial depletion controls: germ-free animals or antibiotic-depleted mice, in which the gut microbiota is removed before wounding, should be included to test whether the axis is required rather than merely associated (138). Two caveats should be stated explicitly: antibiotic cocktails themselves alter cutaneous immunity and delay repair, so depletion must be verified by sequencing and interpreted against an antibiotic-treated control with intact wounding only; and since no study has yet compared type 1 and type 2 diabetic models end-to-end for the gut–liver–wound axis, model selection should be reported as a reasoned choice and validated by showing that the chosen model reproduces the dysbiosis–endotoxemia–hepatic inflammatory phenotype.

### 4.5 Multi-target synergistic intervention and personalized optimization

This level emphasizes the organic integration of different strategies, rather than their simple superposition. The molecular basis for their synergistic effects can be understood from the following perspectives (1): Interaction between SCFAs and the bile acid pathway: SCFAs produced by the gut microbiota may lower the pH of the intestinal lumen and thereby influence bile acid solubility; however, whether this translates into enhanced bile acid reabsorption *in vivo* remains to be directly demonstrated (2). Because the gut microbiota regulates hepatic drug-metabolizing enzymes —cytochrome P450 3A (Cyp3a) expression and activity are markedly reduced in germ-free mice and are normalized upon conventionalization (139, 140) — we hypothesize that, after FMT has restored the gut microbial community, exogenous supplementation with SCFAs or bile acid analogues could accelerate the recovery of metabolic conversion capacity and thereby mitigate dysbiosis-associated drug-metabolism abnormalities. Notably, human-fecal FMT into germ-free mice did not restore hepatic cytochrome P450 3A4 (CYP3A4) expression, indicating that restoration of the microbial community alone may be insufficient (141) (3); Programmable nanodelivery as an enabling tool: nanodelivery is not a separate biological level but a technology deployable at each level of the sequence above. The biological logic remains peripheral-to-core—SCFAs released in the gut to repair the barrier and reduce endotoxemia; FXR agonists delivered via the portal vein to the liver to correct metabolic dysregulation; exosomes released in the wound to drive M2-like polarization—together forming a “gut–liver–wound” three-stage sequential therapy, which should be understood as a working hypothesis rather than an established therapeutic paradigm (132).Carrier chemistry alone does not specify timing, so the response mechanism must match the target phase: in the inflammatory phase, when ROS are high, the wound bed is alkaline, and M1-like macrophages dominate, ROS-labile boronate linkages release M2-promoting cargo earliest while scavenging ROS (92); as ROS and pH normalize, the remaining pro-angiogenic and pro-proliferative payloads support the proliferative phase, and MMP-9–keyed release has been designed to track the inflammation-to-proliferation transition (93); in remodeling, slow release favors collagen maturation. Whether these kinetics overlap the M1-like→M2-like window is empirical: *in vivo* release half-lives should be compared with the inducible nitric oxide synthase-positive (iNOS^+^)→CD206^+^ time course, not inferred from *in vitro* curves. Coupling should be verified with tissue-level readouts in the same animals—granulation tissue thickness, CD31^+^ vessel density, collagen maturity (picrosirius red), and phenotype-resolved cell density (39). A key limitation is stimulus variability: 76.4% of diabetic foot ulcers were alkaline at baseline and pH falls during healing, with a ~0.5-unit center-to-edge gradient within a single wound (142), so a fixed threshold releases early, late, or not at all depending on patient and time point; comparable human ROS data are lacking. Predictability therefore requires patient-specific calibration, dual or self-regulated response systems, and in-wound monitoring.

In the future, if single-cell and integrated multi-omics datasets of diabetic wounds (88, 89) become sufficiently standardized, they could be explored to systematically analyze the temporal contributions and dose–response relationships of each component in combined interventions regarding macrophage phenotypic conversion, and to inform a conceptual “digital twin” framework; however, no prospective study has demonstrated that a digital-twin-guided intervention improves DFU healing. In the near term, however, these technically demanding approaches should be complemented—and can be preceded—by more readily implementable tissue-level validation: immunohistochemical profiling of wound-resident macrophages using CD68 together with iNOS and CD86 for M1-like cells and CD206 together with Arg1 for M2-like cells (143), quantified by H-score and free digital image-analysis platforms (ImageJ/Fiji and equivalents) with observers blinded to group allocation (144, 145). Because circulating LPS is not yet linked to a wound-level dose–response, quantifying the M1-like/M2-like ratio along a defined wound axis (wound edge versus center) at defined postoperative time points offers a direct and actionable test of the pre-programming hypothesis and of its dose–response prediction (146). These approaches remain conceptual at present and require validation in prospective studies.

### 4.6 Limitations and barriers to clinical translation

Several overarching limitations constrain clinical translation. First, evidence gaps are profound: for DFU healing, no intervention has reached large-RCT level; the strongest wound-specific evidence comes from small RCTs of topical probiotics (123) and MSC-derived exosomes (130). FMT, engineered probiotics, engineered exosomes, nanodelivery and digital twins remain preclinical or hypothetical (89). Second, safety concerns include pathogen transmission with FMT, pruritus in 28–51% with FXR agonists (125), biocontainment risks with engineered probiotics, exosome immunogenicity and batch heterogeneity, and nanocarrier accumulation. Third, delivery is limited by wound pH heterogeneity (142), multi-barrier transport along the gut–liver–wound axis, oral probiotic viability (116), exosome targeting and nanocarrier penetration. Fourth, standards remain unmet: exosome isolation and potency assays (107), probiotic strain identity, nanocarrier batch reproducibility, and FMT donor screening. Finally, clinical validation requires multicenter randomized blinded trials with wound-specific endpoints, long-term follow-up, and stratification by microbiome and wound pH.

## 5 Conclusions and perspectives

Dysregulated macrophage function is a central cause of impaired healing in diabetic wounds, and multiple mechanisms—epigenetic regulation, miRNAs, exosomes, and metabolic pathways—are now implicated in macrophage polarization in this setting. The role of the gut–liver axis in diabetic wound repair and its interplay with macrophage regulation nonetheless remain relatively underexplored.

In this Review, we have systematically delineated a conceptual framework in which gut–liver axis dysfunction in diabetes may contribute to a systemic pro-inflammatory “set point” through sustained release of LPS, inflammatory cytokines, and dysregulated metabolites. This chronic inflammatory milieu acts on bone marrow hematopoietic progenitors, inducing persistent epigenetic modifications (e.g., H3K4me3 and H3K27ac enrichment) and metabolic reprogramming (a shift toward glycolysis) that “pre-program” circulating monocytes toward an M1-like-biased phenotype prior to their arrival at the wound site. Upon recruitment to the wound and exposure to local hyperglycemic and hypoxic stimuli, these pre-programmed monocytes are predisposed to lock into the M1-like state, thereby perpetuating chronic inflammation and impairing tissue repair. The intervention framework we propose—gut microbiome remodeling, metabolite supplementation, and macrophage-directed therapy (including exosome-based approaches) as sequential biological levels, integrated through multi-target synergy and implemented by a cross-cutting enabling technology, programmable nano-delivery—provides a systematic roadmap for translating this mechanistic understanding into clinical strategies.

Several limitations should be considered when interpreting the proposed mechanisms linking the gut–liver axis to macrophage dysfunction and diabetic wound healing. Potential confounding factors fall into three main categories. First, medications, including antibiotics, metformin, and proton pump inhibitors, can alter gut microbial composition and metabolic activity, potentially complicating the distinction between disease-associated and treatment-associated microbial changes (147). Second, lifestyle and host-related factors, including dietary patterns, age, and geographical differences, may contribute to variation in microbial communities, metabolite exposure, and immune responses, thereby limiting comparisons across study populations and the generalizability of the proposed mechanisms (148). Third, obesity and comorbid conditions, including liver disease, infection, and vascular insufficiency, may influence microbial ecology, systemic or local inflammation, macrophage function, and tissue repair through overlapping or independent pathways. Collectively, these factors may confound the observed associations among gut microbial alterations, macrophage phenotypes, and wound-healing outcomes. Consequently, macrophage dysfunction and shifts between M1-like and M2-like phenotypes cannot be attributed specifically to the gut–liver axis without adequately accounting for these alternative influences. Moreover, the M1/M2 framework represents a simplified description of the heterogeneous macrophage states present in wounds (149, 150). Future studies should incorporate detailed medication and clinical histories, appropriate cohort stratification and multivariable adjustment, and controlled mechanistic experiments to distinguish gut–liver-axis-mediated effects from confounding influences and establish their causal relevance to diabetic wound healing.

Future work also should further elucidate how the gut–liver axis fine-tunes macrophage function through the microbiota–host metabolite–immune cell axis, and should seek to develop therapeutic strategies targeting the gut microbiota or its metabolites to promote diabetic wound healing. Integrating modulation of the gut–liver axis with existing macrophage-targeted strategies may offer new therapeutic concepts and targets for diabetic wound repair; however, these concepts remain speculative pending causal validation, and any benefit to prognosis and quality of life in patients with diabetes remains to be demonstrated. A major current knowledge gap is whether, and by what mechanisms, gut–liver axis–derived metabolites or exosomes traverse vascular barriers, enter wound tissue, and act directly on macrophages; *in vivo* tracing combined with single-cell spatial transcriptomics should determine whether a complete “gut–liver–blood–wound” signaling axis indeed exists.

To validate the core “systemic pre-programming” hypothesis in the near term, three experiments should be prioritized, each prespecifying the same histomorphometric endpoint battery so that results are directly comparable across designs rather than reporting molecular readouts alone. This battery comprises wound closure rate; re-epithelialization distance on hematoxylin and eosin–stained sections; granulation tissue thickness; and phenotype-resolved inflammatory cell density—lymphocyte antigen 6G-positive (Ly6G^+^) neutrophils, F4/80^+^ macrophages, and iNOS^+^ or CD86^+^ (M1-like) versus CD206^+^ or Arg1^+^ (M2-like) cells per high-power field—quantified by H-score or digital image analysis (ImageJ/Fiji and equivalents) at the wound edge and the wound center at defined time points (39) (1): bone marrow transplantation in both directions (diabetic → wild-type and wild-type → diabetic), to test whether the pre-programmed bone marrow compartment is sufficient to transfer the wound healing deficit (2, 131) (2); paired portal vein and peripheral blood sampling, to establish dose–response relationships between gut-derived signals (LPS, secondary BAs, and SCFAs) and the same tissue-level endpoints in the same animals; and (3) FMT combined with macrophage adoptive transfer, using fluorescently labeled donor macrophages, to determine whether gut microbiota restoration improves wound healing through altered macrophage function (110, 151). All three designs should use randomized allocation, powered sample sizes, and blinded measurement. These experiments could help transform the current correlative evidence into a causal understanding of how the gut–liver axis pre-programs wound macrophages, and could provide the mechanistic basis for combined microbiota- and macrophage-directed therapies in diabetic wound repair.
