# Supplementary material for: Regulation of diabetic wound healing by the gut–liver axis via immunometabolic reprogramming of macrophages: from systemic pre-programming to targeted therapy
Source: Front Immunol. 2026 Oct 30;17:1991888. doi: 10.3389/fimmu.2026.1991888 (PMC13760828; doi:10.3389/fimmu.2026.1991888)
Supplement: Supplementary file 1 [file Table1.docx]

**Supplementary Table 1. Detailed evidence status of proposed interventions targeting the gut–liver–macrophage axis in diabetic wound healing.**

| **Strategy category (axis level)** | **Intervention** | **Primary target(s)** | **Delivery route** | **Study model / population** | **Evidence level** | **Clinical status / DFU relevance** | **Main findings (with references)** |
| --- | --- | --- | --- | --- | --- | --- | --- |
| **Gut microbiota** | Probiotics / synbiotics | Gut microbiota | Oral; topical | Diabetic mice (db/db or HFD+STZ); double-blind small RCT in DFU (topical probiotic in soybean concentrate) | Early clinical | Small RCT completed for DFU | Restores intestinal barrier integrity, reduces circulating LPS, and ameliorates systemic low-grade inflammation. A DFU RCT (topical probiotic) accelerated complete healing (mean 89.6 vs. 165.9 days; P = 0.012) and was safe and well tolerated (37, 113, 123, 137). |
| **Gut microbiota** | FMT | Gut microbiota | Oral or colonoscopic | Diabetic mice (HFD+STZ); RCTs in obese T2D and obesity with insulin resistance | Preclinical | Early clinical in T2D/obesity; no DFU trials; mixed clinical results | Improves glycemic homeostasis, attenuates systemic inflammation, and reshapes gut microbial architecture. A 24-week double-blind RCT in obese T2D reported increased lean-donor microbiota engraftment and butyrate-producing bacteria, reduced total and LDL cholesterol and liver stiffness, without serious adverse events; an oral FMT capsule RCT in obesity with insulin resistance showed engraftment but no significant improvement in insulin sensitivity (110, 120, 121). |
| **Gut metabolites** | SCFA supplementation | Macrophage metabolic reprogramming; intestinal barrier | Oral | db/db mice | Preclinical | No DFU trials | Promotes M2-like macrophage polarization, enhances intestinal barrier function, and suppresses inflammatory responses. Butyrate acts as an HDAC inhibitor, restoring histone acetylation, reprogramming the macrophage transcriptome, and enhancing phagocytosis and coordinated inflammatory responses (64, 67, 83, 111). |
| **Liver / bile acid** | OCA / FXR agonists | Bile acid signaling (FXR/TGR5 pathway) | Oral | MASH mouse models (OCA); db/db or HFD+STZ diabetic wound models (extrapolation) | Preclinical | Phase 3 in MASH; no DFU trials; requires dose selection and monitoring | Inhibits the NF-κB inflammatory cascade, regulates bile acid metabolism, and attenuates hepatic and systemic inflammation. In the phase 3 REGENERATE trial (MASH), pruritus was the most common adverse event (placebo 19% vs. OCA 10 mg 28% vs. 25 mg 51%); serious adverse events were similar across groups, and hepatic, renal, and cardiovascular adjudicated events remained low and comparable (112, 114, 124, 125, 126). |
| **Liver / bile acid** | UDCA | Bile acid signaling (context-dependent FXR modulation; not a classical FXR agonist) | Oral | Human morbid obesity (clinical study); in vitro and in vivo models | Preclinical | No targeted DFU trials | Modulates FXR/TGR5-mediated bile acid signaling in a context-dependent manner, suppresses inflammatory responses, and regulates lipid metabolism (50). |
| **Systemic / wound macrophage** | MSC-derived exosomes | Wound macrophages; angiogenic processes | Local (wound); intravenous | Diabetic rats (STZ-induced full-thickness excisional wound model); RCT in 110 patients with persistent DFU (topical Wharton's jelly MSC-derived exosomes plus standard of care) | Early clinical | RCT (n = 110) completed for DFU | Drives M2-like macrophage polarization, promotes angiogenesis, and accelerates diabetic wound healing. In an RCT of 110 patients with persistent DFU, topical Wharton's jelly MSC-derived exosomes plus standard of care increased complete healing and shortened mean time to complete epithelialization (approximately 6 weeks vs. 20 weeks in controls) (73, 91, 130). |
| **Systemic / wound macrophage** | Engineered exosomes (miRNA-loaded) | Wound macrophages; angiogenesis; re-epithelialization | Local (wound); intravenous | Diabetic mice (db/db or STZ-induced) | Preclinical | No DFU trials | Enhances angiogenic activity, regulates macrophage polarization, and promotes tissue repair via PI3K/Akt, NF-κB, MAPK, TGF-β/Smad, and HIF-1α/VEGF signaling (4, 23, 71, 72). |
| **Systemic / wound macrophage** | Systemic pre-programming hypothesis | Hematopoietic stem/progenitor cells and circulating monocyte precursors | Not applicable | None (theoretical hypothesis) | Hypothetical | No experimental validation | Proposes that gut–liver-derived LPS, cytokines, and exosomes (via TLR4/NF-κB) pre-program bone-marrow monocytes toward a pro-inflammatory M1-like bias before wound homing, mediated by persistent histone marks (H3K4me3, H3K27ac) and aerobic glycolysis. To be tested by (1) bidirectional bone marrow transplantation, (2) paired portal vein and peripheral blood sampling, and (3) FMT combined with macrophage adoptive transfer (2, 52, 54, 110, 131). |
| **Wound-local delivery** | Butyrate-loaded microneedles | Wound-site macrophages | Topical (microneedle) | db/db mice; topical microneedle delivery | Preclinical | No DFU trials | Enhances M2 polarization of local wound macrophages, promotes angiogenesis, and accelerates wound closure (14, 63, 111). |
| **Cross-cutting enabling technology** | Smart responsive nano-delivery system | Multisite (intestine, liver, wound bed) | Multisite (intestine, liver, wound) | Diabetic mice or rats (db/db or STZ-induced full-thickness excisional wound model) | Preclinical | No RCT in DFU; early-phase biologic delivery only | Enables pH/ROS-sensitive, spatiotemporally controlled drug release with ROS-labile boronate linkages and MMP-9-keyed release. A key limitation is stimulus variability: 76.4% of DFU are alkaline at baseline, pH falls during healing, with a ~0.5-unit center-to-edge gradient; comparable human ROS data are lacking, requiring patient-specific calibration, dual/self-regulated responses, and in-wound monitoring (92, 93, 115, 132, 142). |
| **Cross-cutting enabling technology** | Multi-target synergistic therapy / personalised optimisation | SCFA–bile acid interaction; gut–liver–wound sequential axis | Multisite | None (conceptual framework) | Hypothetical | No experimental validation | Envisages a gut–liver–wound three-stage sequential therapy combining FMT with SCFA or bile acid analogues and programmable nano-delivery as an enabling tool, guided by digital-twin models built on single-cell and multi-omics data with tissue-level validation. A key limitation is that human-fecal FMT into germ-free mice did not restore hepatic CYP3A4 expression, indicating that restoring the microbial community alone may be insufficient (132, 139, 140, 141, 142). |

**Alt text:** Within each axis level, interventions are ordered by evidence level (Early clinical → Preclinical → Hypothetical). Wound-local delivery and cross-cutting enabling technology are delivery/technology layers rather than independent biological levels, and are therefore placed after the biological axis levels. Reference numbers are given in parentheses at the end of Main findings.

**Note:** DFU, diabetic foot ulcer; FMT, fecal microbiota transplantation; FXR, farnesoid X receptor; HDAC, histone deacetylase; HFD+STZ, high-fat diet plus streptozotocin; HIF-1α, hypoxia-inducible factor 1 alpha; LDL, low-density lipoprotein; LPS, lipopolysaccharide; MASH, metabolic dysfunction-associated steatohepatitis; MMP-9, matrix metalloproteinase 9; MSC, mesenchymal stem cell; NF-κB, nuclear factor-kappa B; OCA, obeticholic acid; RCT, randomized controlled trial; ROS, reactive oxygen species; SCFA, short-chain fatty acid; T2D, type 2 diabetes; TGR5, G protein–coupled bile acid receptor 1; TLR4, Toll-like receptor 4; UDCA, ursodeoxycholic acid. Evidence levels: Preclinical = animal or in vitro studies; Early clinical = Phase 1/2 or small RCT; Clinical = Phase 3 or larger RCT; Hypothetical = no experimental validation.
